# PRMT6-mediated ADMA promotes p62 phase separation to form a negative feedback loop in ferroptosis

**DOI:** 10.7150/thno.94789

**Published:** 2024-07-02

**Authors:** Lifeng Feng, Lini Chen, Weikai Wang, Qi Wei, Minqiang Chen, Xin Jiang, Shiman Hu, Yuchen Wu, Lian Duan, Liyuan Zhu, Xian Wang, Hongchuan Jin

**Affiliations:** 1 Department of Medical Oncology, Zhejiang Key Laboratory of Multi-omics Precision Diagnosis and Treatment of Liver Diseases, Cancer Center of Zhejiang University, Sir Run Run Shaw Hospital, School of Medicine, Zhejiang University, Hangzhou, Zhejiang, China.; 2 Department of Hepatobiliary and Pancreatic Surgery, Jinhua Municipal Central Hospital, Jinhua, Zhejiang, China.; 3 Department of General Surgery, Sir Run Run Shaw Hospital, School of Medicine, Zhejiang University, Hangzhou, Zhejiang, China.

**Keywords:** ferroptosis, p62, ADMA, PRMT6, phase separation

## Abstract

**Purpose:** Due to intrinsic defensive response, ferroptosis-activating targeted therapy fails to achieve satisfactory clinical benefits. Though p62-Keap1-Nrf2 axis is activated to form a negative feedback loop during ferroptosis induction, how p62 is activated remains largely unknown.

**Methods:** MTS assay was applied to measure cell growth. Lipid ROS was detected with C11-BODIPY reagent by flow cytometer. Quantitative real-time PCR (qPCR) and western blotting were performed to determine mRNA and protein level. Immunofluorescence (IF) was performed to examine the distribution of proteins. Fluorescence recovery after photobleaching (FRAP) was adopted to evaluate p62 phase separation. Immunoprecipitation (IP), co-IP and Proximal ligation assay (PLA) were performed to detected protein posttranslational modifications and protein-protein interactions. Tumor xenograft model was employed to inspect *in vivo* growth of pancreatic cancer cells.

**Results:** Upon ferroptosis induction, Nuclear Factor E2 Related Factor 2 (Nrf2) protein and its downstream genes such as HMOX1 and NQO1 were upregulated. Knockdown of p62 significantly reversed Nrf2 upregulation and Keap1 decrease after ferroptosis induction. Knockdown of either p62 or Nrf2 remarkably sensitized ferroptosis induction. Due to augmented p62 phase separation, formation of p62 bodies were increased to recruit Keap1 after ferroptosis induction. Protein arginine methyltransferase 6 (PRMT6) mediated asymmetric dimethylarginine (ADMA) of p62 to increase its oligomerization, promoting p62 phase separation and p62 body formation. Knockdown of p62 or PRMT6 notably sensitized pancreatic cancer cells to ferroptosis both *in vitro* and *in vivo* through suppressing Nrf2 signaling.

**Conclusion:** During ferroptosis induction, PRMT6 mediated p62 ADMA to promote its phase separation, sequestering Keap1 to activate Nrf2 signaling and inhibit ferroptosis. Therefore, targeting PRMT6-mediated p62 ADMA could be a new option to sensitize ferroptosis for cancer treatment.

## Introduction

As a unique modality of programmed cell death, ferroptosis is driven by accumulating lipid peroxidation largely dependent on cellular free iron 1, 2. Recently, targeted therapy to activate ferroptosis has been recommended as an effective strategy for cancer treatment. However, administration of ferroptosis-inducing drugs such as sorafenib alone failed to achieve satisfactory clinical benefits, due to intrinsic or acquired tolerance developed soon or later 2, 3. Therefore, combination strategies based on the understanding of the defensive mechanisms might be promising solutions for ferroptosis-inducing cancer targeted therapies.

The homeostasis of lipid peroxidation plays a very important role in the regulation of ferroptosis. As one of the dominant defense mechanisms for ferroptosis, Glutathione peroxidase 4 (GPX4) detoxifies lipid peroxides by utilizing reduced glutathione 4. Therefore, ferroptosis could be effectively augmented by inhibition of GPX4, as well as suppression of solute carrier family 7 member 11 (SLC7A11), the catalytic subunit of system X_C_^-^ that blocks the importing of extracellular cysteine essential for glutathione synthesis 4.

Recent advances unveiled the Nuclear Factor E2 Related Factor 2 (Nrf2) signaling as the primary mechanism for neutralizing ferroptosis 5, 6. For instance, Nrf2 could transactivate the light chain and heavy chain of ferritin, the main iron storage protein that reducing cellular free iron 7, 8, and SLC7A11 that facilitating glutathione synthesis 9. Additionally, antioxidant genes such as NQO1 and HMOX1 are also Nrf2 downstream genes that could reduce lipid peroxides 10, 11. SQSTM1/p62 (hereafter referred as p62) could facilitate the autophagic degradation of the E3 ubiquitin ligase Keap1, thus upregulating Nrf2 protein level by protecting it from Keap1-dependent proteasomal degradation 12. This p62-Keap1-Nrf2 axis is activated to form a negative feedback loop after ferroptosis induction 13-15. Therefore, it would be important for targeting this negative feedback loop to ensure the potential clinical efficacy of ferroptosis-inducing drugs. Unfortunately, how p62 is activated to dissociate keap1 from Nrf2 upon ferroptosis induction is not understood yet.

Arginine methylation, including mono-methylarginine (MMA), asymmetric dimethylarginine (ADMA), and symmetric dimethylarginine (SDMA), is a protein post-translational modification catalyzed by arginine methyltransferases (PRMTs). Recent advances revealed that protein arginine methylation modifications play critical roles in regulating protein function and stability 16, 17. While all PRMTs could catalyze the formation of MMA as an intermediate, ADMA is catalyzed by type I family of PRMTs including PRMT 6. In the current study, we figured out that ferroptosis inducers promoted PRMT6-mediated ADMA to enhance p62 phase separation via increasing its oligomerization, resulting in the activation of p62-Keap1-Nrf2 axis to attenuate ferroptosis. Therefore, inhibition of PRMT6-mediated p62 ADMA could be a new option to sensitize ferroptosis for cancer treatment.

## Materials and Methods

### Cells, chemicals and antibodies

Hela and Human pancreatic cancer cell lines including MIAPACA-II, BxPC3, PANC-1 etc., were purchased from Cell Bank of the Typical Culture Preservation Committee, Chinese Academy of Sciences (Shanghai, China). Human liver and colorectal cancer cell lines used were the same as previous reports 18, 19. All cells were maintained with recommended medium supplemented with 10% FBS.

The chemicals used were listed as follows: RSL3 (HY-100218A), Liproxstatin-1 (HY-12726) from MedChemExpress LLC (Shanghai, China); ML162 (S4452), erastin (S7242), MS049 (S8147), EPZ020411 (S7820) from Selleck (Shanghai, China), DMSO (D8418); AdoX (A7154), 1, 6-hexanediol (240117) from Sigma-Aldrich (Shanghai, China).

The antibodies used were as follows: anti-p62 (PM045 and m162-3) from MBL CO., LTD (Beijing, China); anti-p-p62 (S403) (GTX128171) from GeneTex (CA, USA); anti-Nrf2 (16396-1-AP), anti-Keap1 (10503-2-AP) and anti-JMJD6 (16476-1-AP) from Proteintech Group (Wuhan, China); anti-ADMA (13522), anti-SDMA (13222), anti-MMA (8015), anti-Myc (2276), anti-beta-actin (8457) from Cell Signaling Technology (Shanghai, China); anti-PRMT6 (A7814) from ABclonal Technology (Wuhan, China); anti-Flag (F1804 and A2220) from Merck (Shanghai, China); anti-GFP (AB290), anti-acetyl Lysine (ab21623) from Abcam (Shanghai, China); GFP agarose (NBS01A) from HUABIO (Hangzhou, China).

### Plasmids construction and transfection

The full length of Flag-p62, its truncations and deletions used were the same as previous report 18, GFP tagged p62 (GFP-p62) and its mutants were constructed with ClonExpress MultiS One Step Cloning Kit (C113) from Vazyme (Nanjing, China), using Flag-p62 and mutants as templates. Myc-PRMT1, 2, 3, 4, 6 and Flag-KDMs (KDM3A, 4A, 4E, 5C and JMJD6) were kindly provided by Dr. Jia Zhou at Sir Run Run Shaw Hospital, School of Medicine, Zhejiang University. p62 arginine residue 183 and 217 (R183 and R217) site mutation, as well as enzyme inactive PRMT6 mutant (PRMT6mt, aas86-88: VLD mutated to KLA) were introduced with the QuickChange II Site-Directed Mutagenesis Kit (Aligent, Beijing, China). Myc-PRMT6 truncations and mCherry-PRMT6wt/mt were constructed using Myc-PRMT6wt/mt as templates. The lentiviral Flag-p62 and its 2RK mutant were ligated into pLVX vector. And all the shRNA-resistant p62 constructs containing nonsense mutations of C1017T, T1020C, and G1023A as previously reported 20, were constructed using the QuickChange II Site-Directed Mutagenesis Kit. Then, cells were infected with lentivirus and selected by puromycin.

### SiRNA/shRNA design, construction

Nrf2, p62, PRMT6 targeted siRNAs and those siRNAs targeting KDMs were synthesized by Gene Pharma Company (Shanghai, China). Lipofectamine^TM^ RNAiMAX transfection reagent (Thermo Fisher Scientific, Shanghai, China) was used for siRNA transfection following the instruction. PRMT6 and p62 shRNAs (shPRMT6 and shp62) were constructed via ligation of targeting oligonucleotides into the Xho I/Mlu I digested pGIPZ vector. And MIAPACA-II cells were infected with shp62 or shPRMT6 lentivirus, and then selected by puromycin. The sequence detail of siRNAs and shRNAs used in this study were listed in Supplemental Table.

### RNA extraction, reverse transcription and quantitative real-time PCR (qPCR)

Trizol reagent (CW0580S, Cowin Bio., Taizhou, China) was used to extract total RNA following the manufacturer's instruction, and RNA concentrations were determined with the NanoDrop 2000c (Thermo Fisher Scientific). Up to 2 μg total RNA was reverse transcribed with the High-Capacity cDNA Reverse Transcription Kit (Thermo Fisher Scientific), and the quantitative real-time PCR (qPCR) was performed using Ultra SYBR Mixture (CW0957M, Cowin Bio.) with Light Cycler 480 II system (Roche, Shanghai, China). GAPDH was adopted as the internal control for normalization. Three biological replicates of each sample were employed, and the results were shown as mean ± SD. The details of the primers used were all listed in Supplemental Table.

### CRISPR-Cas9 knockout cell line construction

p62 knockout (KO) cell line was generated by the CRISPR-Cas9 system. Human p62 targeting single guide RNAs (sgRNAs) were designed with CRISPR Designer (http://crispr.mit.edu/), and the nucleotide sequences targeting p62 are as follows: 5ʹ-CACCGACCGTGAAGGCCTACCTTCT-3ʹ and 5ʹ-AAACAGAAGGTAGGCCTTCACGGTC-3ʹ. The synthesized sgRNAs were annealed and cloned into pX330 vector, then packaging into lentivirus. Hela cells were infected, selected by puromycin and seeded in 96-well plates for single colony isolation. The knockout of p62 was validated by western blotting.

### MTS assay

Cells were seeded in 96-well plates at a density of 3 × 10^3^ cells per well in the appropriate medium and treated as indicated. After the treatment, cell viability was assessed by MTS regents (G1111, Promega, Beijing, China) according to the manufacturer's instruction. The absorbance of each well at 490 nm was measured with a BioTek Gen5 (Aligent) microplate spectral photometer. Three biological replicates were employed, and the results were shown as mean ± SD.

### Immunofluorescence (IF) and 1,6-hexanediol (1,6-HD) treatment

Cells seeded on coverslips were incubated with ferroptosis inducers or transfected with plasmids as indicated, then fixed with pre-cold methanol for 10 mins, permeabilized with 0.25% Triton X-100 (diluted in 1 × PBS) for 10 mins, and blocked with 3% bovine serum albumin (BSA, diluted in 1 × PBS) for 30 mins. For the 1,6-hexanediol (1,6-HD) treatment, 5% 1,6-HD was added for another 15 mins after permeabilization as needed. The samples were further incubated with relevant primary antibodies at 4 °C overnight. And the coverslips were washed with PBS-T (1 × PBS with 0.1% Tween-20) for three times, incubated with appropriate fluorescence dyes conjugated secondary antibodies (Thermo Fisher Scientific) for 1 h at 37 °C, then washed and sealed with VECTASHIELD Medium with DAPI (H1200, Vector Laboratories). The represent images were captured by a confocal microscopy (Olympus, Japan). And the numbers of p62 body were counted in 5 independent cells per sample, the results were shown as mean ± SD.

### Fluorescence recovery after photobleaching (FRAP)

Hela or p62 knockout (p62-KO) cells were transfected with GFP-p62-WT or its mutants, together with or without mCherry-PRMT6. After indicated treatments, the live cells were sent for FRAP experiments using an Olympus confocal microscopy. The GFP-p62 bodies were bleached for 2 s with 100% laser intensity at 488 nm, and the recovery of fluorescence was recorded for indicated times.

### Co-Immunoprecipitation (Co-IP) and Western blotting

Briefly, for co-IP, the harvested cells with indicated treatment or transfection were lysed with Triton buffer (50 mM Tris-HCl pH 7.4, 150 mM NaCl, 0.5% Triton-X-100) or Radio immunoprecipitation assay (RIPA) buffer (P0013D, Beyotime, Shanghai, China) supplemented with protease inhibitor cocktail (B14001, Selleck), then quantitated by BCA protein assay kit (P0010, Beyotime). 1 mg total cell lysate was immunoprecipitated with anti-p62 or indicated antibody-conjugated agarose, immuno-complex was washed, boiled and subjected to western blotting.

For western blotting, samples were resolved by SDS-PAGE, transferred to PVDF membrane and incubated with the primary antibodies at 4 °C overnight. The membranes were then washed with TBS-T (0.1% of Tween-20 in TBS) and incubated with relevant HRP-conjugated second antibodies (Jackson Immuno Research, USA). Finally, the membranes were tested with enhanced chemiluminescence (FD8030, Fudebio, Hangzhou, China), and pictures were captured with Amersham Imager 600 system (GE Healthcare Life Sciences, Shanghai, China).

### Biochemical fractionation

The detergent soluble-insoluble fractions were performed as previously reported 21, 22. Generally, Hela cells with indicated treatment such as ferroptosis inducers incubation were harvested and lysed in ice-cold 0.5% NP40 lysis buffer containing protease inhibitors (B14001, Selleck, Shanghai, China) for 30 min. The cell lysates were then centrifuged at 14000 rpm (4 °C) for 30 min. The supernatant fraction was transferred to a new 1.5 mL tube, and 5 × SDS loading buffer was added. And the precipitate was washed once with 0.5% NP40 lysis buffer, resuspended with 1 × SDS loading buffer. Finally, soluble and insoluble fractions were boiled and subjected to western blotting.

### Proximal ligation assay (PLA)

Proximal ligation assay (PLA) was performed with Duolink^®^ In Situ Red Starter Kit (DUO92101, Sigma-Aldirich). Hela or MIAPACA-II Cells were seeded on coverslips overnight, and incubated with ferroptosis inducers for 12 h. Cells were fixed and permeabilized accordingly, and then step by step following the instructions, including blocking, primary antibodies and probes incubation, ligation and amplification. Finally, the coverslips were sealed with DuolinkR PLA Mounting Medium with DAPI, and the photos were captured by an Olympus confocal microscopy.

### Lipid ROS analysis

Cells were seeded in 6-well plates one day before treatment. After incubated with reagents as indicted for 48 h, cells were treated with 5 μM C11-BODIPY (D3861, Thermo Fisher Scientific) for 30 min in the dark. Then, the cells were harvested, washed twice with 1 × PBS, resuspended in 500 μL 1 × PBS and analyzed by BD FACSCalibur™ flow cytometer (BD Biosciences, Shanghai, China) with a 488 nm laser. The percentage of relative lipid ROS was normalized to the control group.

### Mice xenograft model

Animal studies were reviewed and approved by the Ethics Committee for Animal Studies of Sir Run Run Shaw Hospital, School of Medicine, Zhejiang University. To generate mice subcutaneous tumors, 5 × 10^6^ MIAPACA-II control (shNC), stably p62 (shp62) or PRMT6 (shPRMT6) knockdown cells were resuspended with 0.1 mL 1 × PBS and subcutaneously injected into 4-week-old male BALB/c nude mice (n = 6 per group), which were obtained from Shanghai Laboratory Animal Center and housed in the laboratory-animal research center of Sir Run Run Shaw Hospital, School of Medicine, Zhejiang University. Five days later, the mice were treated with RSL3 (10 mg/kg intraperitoneally, every other day). Tumor volume was monitored using Vernier calipers every other day and was calculated with the following formula: 0.5 × length × width^2^. After 10 days treatment, all mice were sacrificed, and tumors were collected, measured, and weighted, fixed in 4% paraformaldehyde for IHC analysis.

### Immunohistochemistry (IHC)

Tissue sections were deparaffinized in xylene and rehydrated in alcohol, following which endogenous peroxidase was blocked with 3% hydrogen peroxide for 5 min. And after that, antigen retrieval was achieved using a microwave and 0.01 M sodium citrate buffer (pH 6.0). Subsequently, the sections were probed with diluted primary antibodies at room temperature for 1 h. After the binding of HRP-conjugated secondary antibodies for 40 min in the dark at room temperature, the slides were incubated with diaminobenzidine (DAB) and counterstained with haematoxylin. The IHC images were acquired with a polarized light microscope (Nikon, Eclipse 80i).

### Statistical analysis

Statistical analysis was performed with student's t-test, and results were shown as mean ± SD. P value < 0.05 was considered as statistically significant in all experiments ('*' as presented in the figures).

## Results

### Phase separation of p62 inactivated ferroptosis by stabilizing Nrf2 protein

**Figure 1:**
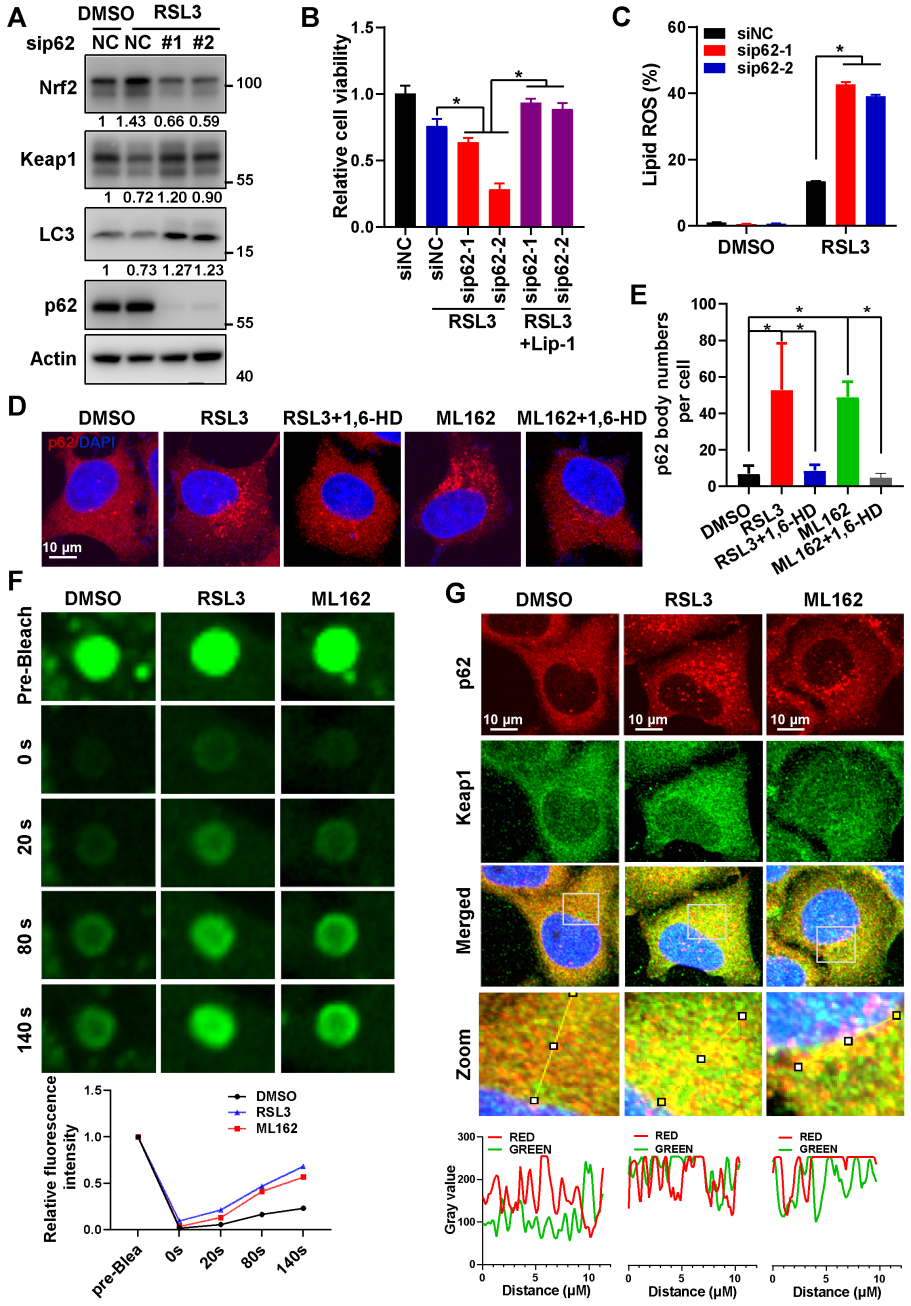
** Phase separation of p62 inactivated ferroptosis by stabilizing Nrf2 protein.** A. The protein level of Nrf2, Keap1 and p62 in Hela cells with p62 knockdown and 3 μM RSL3 treatment for 24 h, were determined by western blotting. Actin was used as a loading control. And relative quantified protein level of Nrf2, Keap1 and LC3-II normalized to Actin were shown. B. After p62 knockdown, Hela cells were dealt with RSL3 for 48 h respectively, together with or without liproxstatin 1 (Lip-1), and cell viability was measured with MTS assay. C. After p62 knockdown, Hela cells were treated with RSL3 for 24 h, lipid ROS was detected with C11-BODIPY reagent by flow cytometer. D. Hela cells cultured with 3 μM RSL3 or 2 μM ML162 for 12 h, were fixed and then treated with or without 5% 1,6-Hexanediol (1,6-HD), p62 distribution was measured by immunofluorescence (IF). E. p62 phase separation in Hela cells with GFP-p62 transfection, with RSL3 (3 μM) or ML162 (2 μM) for 12 h incubation, was determined by fluorescence recovery after photobleaching (FRAP). And the relative quantified fluorescence intensity was shown. F. Co-localization of p62 and Keap1 in Hela cells with RSL3 (3 μM) or ML162 (2 μM) 12 h incubation was detected by IF. The co-localization of p62 and Keap1 in the indicated area was analyzed by Image J. The scale bar is 10 μm.

In consistence with previous reports, ferroptosis inducers RSL3 and ML162 dose-dependently upregulated Nrf2 protein level and nucleus accumulation in Hela cells (Figure S1A-B). And knockdown of Nrf2 could downregulated Nrf2 downstream genes such as HMOX1 and NQO1 upon ferroptosis induction (Figure S1C-D), increased the cytotoxicity of ferroptosis inducers (RSL3, ML162 and Erastin) (Figure S1E-G). As previously reported, ferroptosis inducers reduced Keap1 protein level, and activated autophagy (Figure 1A, Figure S2A-B). In addition, knockdown of p62 during ferroptosis activation reduced the upregulation of Nrf2 and its downstream targeted genes, and increased Keap1 protein level on the contrary (Figure 1A, Figure S2A-E). Not to our surprise, p62 knockdown augmented the cytotoxicity of ferroptosis inducers, which could be largely reversed by ferroptosis inhibitor liproxstatin 1 (Lip-1) (Figure 1B, Figure S2F-G). Moreover, p62 knockdown amplified the lipid ROS level increased upon ferroptosis induction (Figure 1C and Figure S2H). In summary, p62 is important to inactivate ferroptosis by upregulating Nrf2 signaling.

In an effort to explore the underlying mechanisms for p62 to inactivate ferroptosis, we found that ferroptosis inducers significantly triggered the formation of p62 bodies, which could be eliminated by 1, 6-hexanediol (1,6-HD) (Figure 1D-E and Figure S3A). Meanwhile, insoluble p62 fraction increased and soluble p62 decreased under ferroptosis inducers treatment, which further indicated p62 accumulation during ferroptosis induction (Figure S3B). Furthermore, fluorescence recovery after photobleaching (FRAP) revealed that ferroptosis inducers could enhance the recovery of GFP signal after bleaching of p62 body in Hela cells expressing ectopic GFP-p62 (Figure 1F), indicating that these p62 bodies were non-membrane condensation formed by liquid-liquid phase separation of p62. It has been reported that phase separation of p62 was associated with its components such as Keap1 and ubiquitinated cargoes 23, 24. Indeed, Keap1 was recruited into p62 bodies upon ferroptosis induction (Figure 1G and Figure S3C). In summary, the phase separation of p62 was facilitated upon ferroptosis activation to recruit Keap1 into p62 body for subsequent autophagic degradation, thus stabilizing Nrf2 protein to form a negative feedback loop in ferroptosis.

### Ferroptosis inducers promoted R183/R217 ADMA of p62 to increase its phase separation

**Figure 2:**
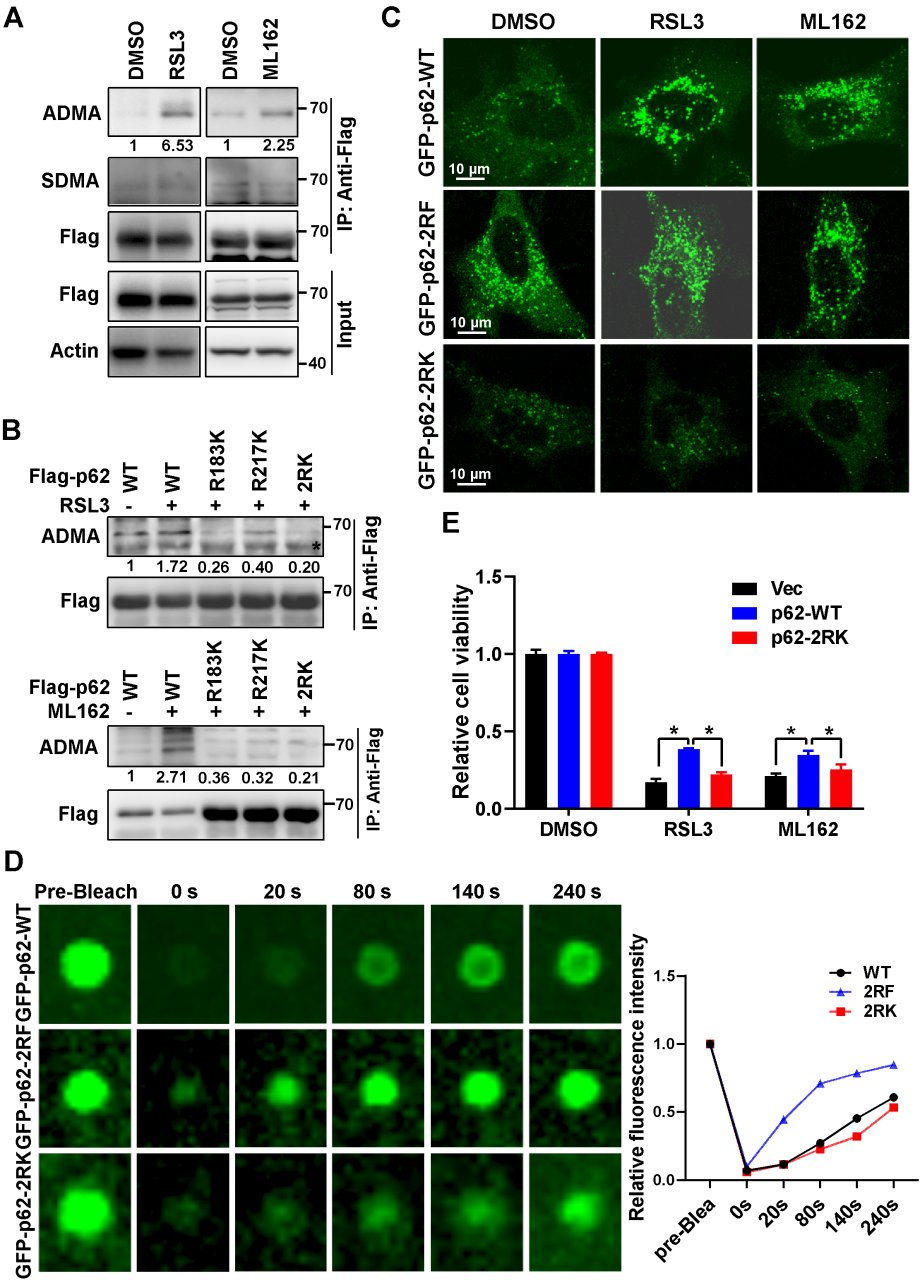
** Ferroptosis inducers promoted R183/R217 ADMA of p62 to increase its phase separation.** A. Flag-p62 expressed Hela cells were incubated with RSL3 (3 μM) or ML162 (2 μM) for 12 h. The lysates were immunoprecipitated with anti-Flag, and immunoblotted with ADMA, SDMA and Flag antibodies. And relative quantified p62 ADMA normalized to immunoprecipitated Flag-p62 was shown. B. Hela cells with wild type (WT) Flag-p62 or indicated mutants Flag-p62-R183K, R217K and R183/217K (2RK) overexpression were cultured with RSL3 (upper) or ML162 (down) for 12 h. The lysates were immunoprecipitated with anti-Flag, and immunoblotted with ADMA and Flag antibodies. C. Flag-p62-WT, 2RK or R183/217F (2RF) mutants were overexpressed in p62-KO cells respectively. After RSL3 (3 μM) or ML162 (2 μM) 12 h treatment, cells were fixed and the GFP signals were explored under a confocal microscopy. D. Phase separation of p62-KO cells with GFP-p62-WT, 2RK or 2RF overexpression was determined by FRAP. And the relative quantified fluorescence intensity was shown. E. Flag-p62-WT, 2RK or control vector (Vec) were transfected into p62-KO cells, and cell viability was measured with MTS assay after cultured with RSL3 (2 μM) or ML162 (1 μM) for 48 h.

It was reported that post-translational modifications such as phosphorylation at serine 403 (S403), or lysine acetylation in the ubiquitin associated (UBA) domain of p62 that increased its binding affinity with ubiquitin chains, could promote p62 phase separation 25-27. However, RSL3 had little effect on S403 phosphorylation and lysine acetylation of p62 (Figure S4A). Mass spectrum analysis indicated that methylation modification on arginine occurred at residues 183 and 217 (R183 and R217) of p62 (Figure S4B). Sequences analysis showed that R183, R217 and their neighboring amino acids are highly conserved among mammals (Figure S4C). Meanwhile, a remarkably elevated asymmetric dimethylarginine (ADMA) rather than symmetric dimethylarginine (SDMA) was observed on p62 after ferroptosis induction (Figure 2A). p62 ADMA induced by ferroptosis inducers was abrogated once R183 and R217 were mutated to lysine (K) in p62 2RK (R183K and R217K) mutant (Figure 2B). To better understand the role of p62 R183/R217 ADMA in ferroptosis, CRISPR-Cas9 technology was applied to construct p62 knockout (p62-KO) Hela cells to exclude the potential impact of endogenous p62. Indeed, p62-KO cells were more sensitive to ferroptosis inducers, accompanied with downregulated Nrf2 and elevated Keap1 protein level (Figure S4D-F). Moreover, ferroptosis inducers could promote the accumulation of GFP-p62. Neither methylation defective GFP-p62-2RK nor methylation mimicking GFP-p62-2RF (arginine mutated to phenylalanine) mutants in p62-KO cells, while GFP-p62-2RF but not GFP-p62-2RK formed plenty of p62 bodies regardless of ferroptosis induction (Figure 2C). FRAP showed that compared to GFP-p62-WT expressed p62-KO cells, the recovery of bleached GFP signal in p62 body was increased in p62-KO cells expressed GFP-p62-2RF, but decreased in GFP-p62-2RK expressed p62 KO cells (Figure 2D). Moreover, reintroduced Flag-p62-WT only but not Flag-p62-2RK could partially attenuate ferroptosis in p62 KO cells (Figure 2E and Figure S4G). Taken together, R183/R217 ADMA of p62 upon ferroptosis induction promote its phase separation to inhibit ferroptosis activation.

### PRMT6-mediated p62 ADMA to promote its phase separation

**Figure 3:**
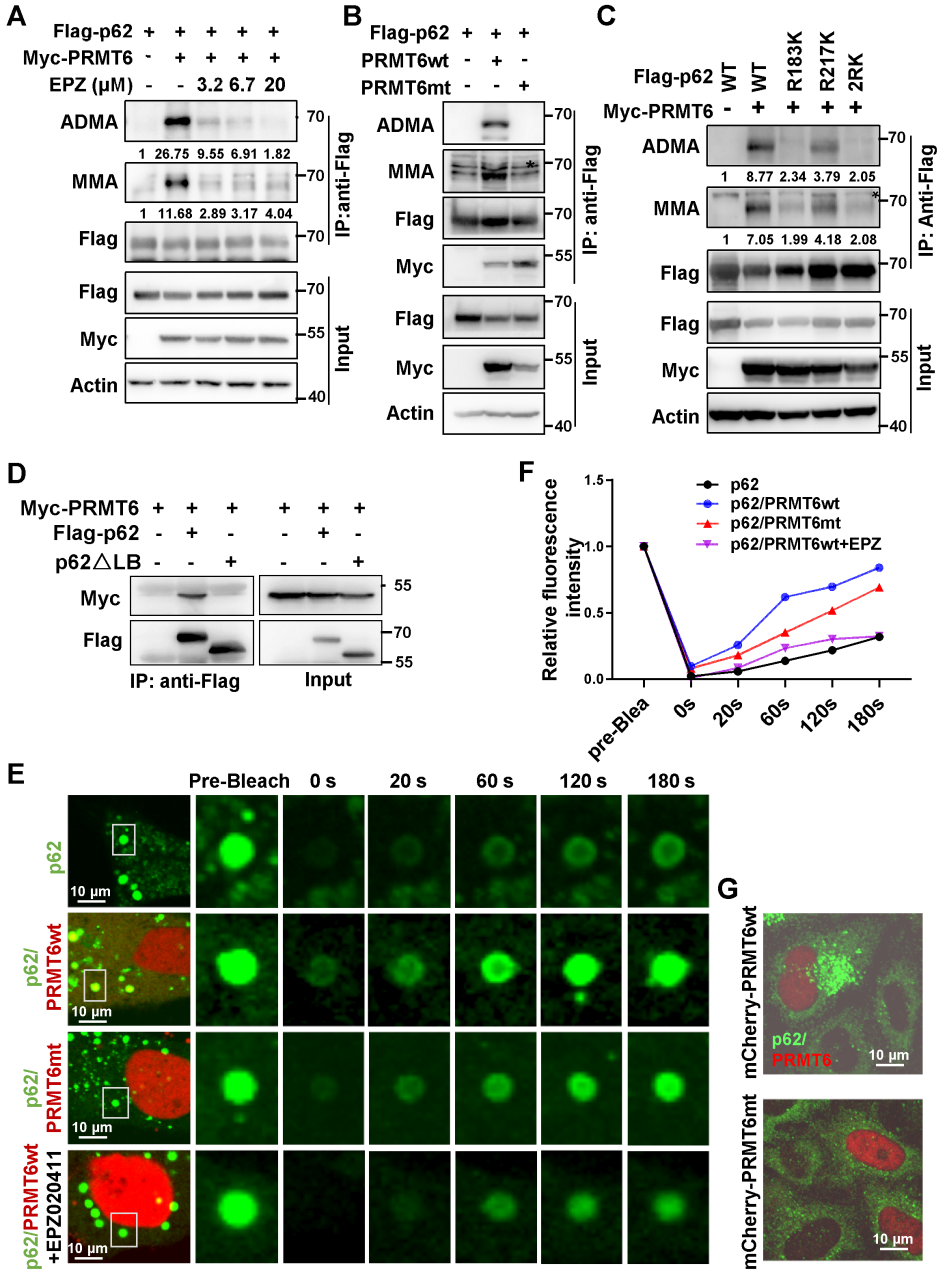
** PRMT6-mediated p62 ADMA to increase its phase separation.** A. HEK293T cells were co-transfected with Flag-p62 and Myc-PRMT6, then overnight treated with indicated concentrations of PRMT6 inhibitor EPZ020411 (EPZ), and Immunoprecipitation (IP) was performed with anti-Flag, followed immunoblotting with ADMA, MMA and Flag antibodies. And relative quantified p62 MMA and ADMA normalized to immunoprecipitated Flag-p62 were shown. B. Immunoprecipitation (IP) of lysates from HEK293T cells co-transfected with Flag-p62 and wild type (wt) Myc-PRMT6 or enzyme inactivated (aas86-88: VLD mutated to KLA) mutant (Myc-PRMT6mt), was performed with anti-Flag, and immunoblotting with ADMA, MMA, Flag and Myc antibodies. C. HEK293T cells were co-transfected with Myc-PRMT6 and Flag-p62-WT, R183K, R217K or 2RK mutants, then IP was performed with anti-Flag, followed by immunoblotting with ADMA, MMA and Flag antibodies. And relative quantified p62 MMA and ADMA normalized to immunoprecipitated Flag-p62 were shown. D. HEK293T cells were co-transfected with Myc-PRMT6 and Flag-p62 or its LB domain deletion (**△**LB) mutant, then co-IP was performed with anti-Flag, followed by immunoblotting with Myc and Flag antibodies. E. Hela cells were transfected with GFP-p62, together with mCherry-PRMT6wt or mCherry-PRMT6mt, with or without EPZ treatment, and FRAP was adopted to analyze the phase separation of p62. F. The relative quantified fluorescence intensity of GFP in 'E' was analyzed by Image J software. G. Hela cells were transfected with mCherry-PRMT6wt or mCherry-PRMT6mt, IF was performed with anti-p62.

To screen potential PRMTs responsible for p62 ADMA, type I family of PRMTs (Myc-PRMT1, 2, 3, 4, 6) that specifically catalyze ADMA were co-expressed with Flag-p62. It turned out that PRMT6 might mediate MMA and ADMA of p62 (Figure S5A). Pan-PRMTs inhibitor AdoX, PRMT4/6 inhibitor MS049, and PRMT6 specific inhibitor EPZ020411 (EPZ) greatly attenuated p62 ADMA (Figure 3A and Figure S5B). In addition, arginine methyltransferase inactive PRMT6 mutant (PRMT6mt) could not upregulate p62 ADMA anymore (Figure 3B). Meanwhile, PRMT6 could barely promote ADMA of p62-2RK mutant (Figure 3C), indicating that PRMT6 mainly mediated p62 ADMA at R183 and R217.

**Figure 4:**
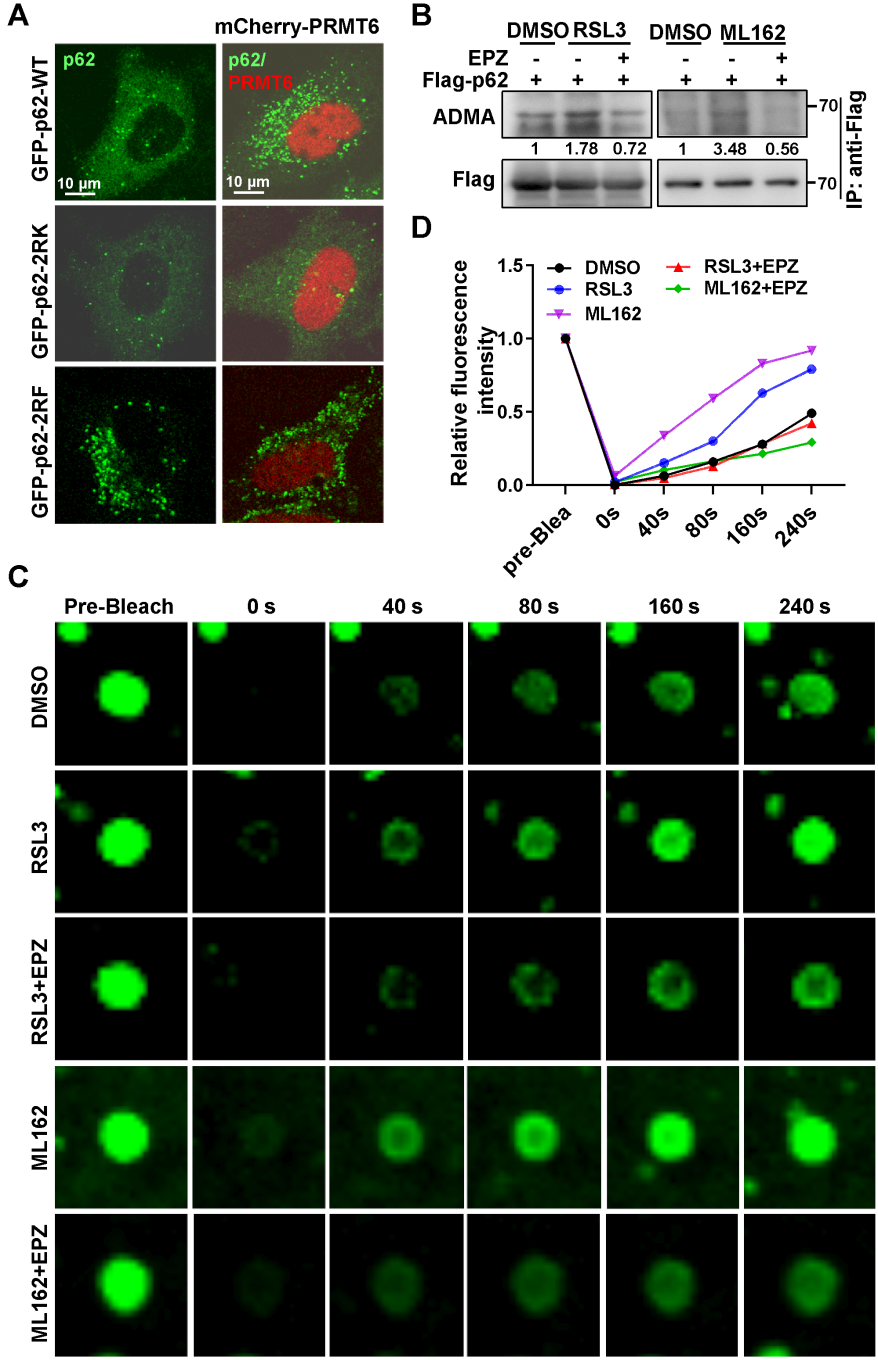
** Ferroptosis inducers augmented PRMT6-dependent ADMA to promote p62 phase separation.** A. Hela cells were transfected with GFP-p62-WT, 2RK or 2RF alone, or together with mCherry-PRMT6, then GFP-p62 and mCherry-PRMT6 signal were examined by a confocal microscopy after fixation. B. Flag-p62 expressed Hela cells were incubated with 3 μM RSL3 (upper) or 2 μM ML162 (down) for 12 h, together with or without EPZ (10 μM), IP was performed with anti-Flag, followed by immunoblotting with ADMA and Flag antibodies. And relative quantified p62 ADMA normalized to immunoprecipitated Flag-p62 was shown. C. GFP-p62 expressed Hela cells were incubated with 3 μM RSL3 or 2 μM ML162 for 12 h, together with or without EPZ (10 μM), and FRAP was applied to analyze the phase separation of GFP-p62. D. The relative quantified fluorescence intensity of GFP in 'C' was analyzed by Image J software.

Truncations of p62 were constructed according to the conserved domain and co-IP experiments verified that it is the LB domain of p62 responsible for the interaction with PRMT6 (Figure 3D and Figure S5C-E). FRAP revealed that mCherry-PRMT6wt, but not mCherry-PRMT6mt, could increase the recovery of GFP signal in p62 body after bleaching in GFP-p62 expressed Hela cells. And PRMT6 inhibitor EPZ retarded the recovery of GFP signal accelerated by PRMT6wt (Figure 3E-F). Next, PRMT6wt, but not PRMT6mt, could trigger the formation of p62 bodies in Hela cells (Figure 3G). Furthermore, mCherry-PRMT6 could promote the accumulation of GFP-p62-WT, but neither GFP-p62-2RK nor GFP-p62-2RF mutants in p62-KO cells (Figure 4A). On the other hand, PRMT6 inhibitor EPZ could suppress p62 ADMA during ferroptosis induction (Figure 4B). Meanwhile, ferroptosis inducers could enhance PRMT6 and p62 interaction to mediate p62 ADMA (Figure S6A-B). In line with these findings, FRAP showed that EPZ significantly retarded GFP signal recovery in GFP-p62 expressed Hela cells after ferroptosis induction (Figure 4C-D).

Besides, previously reported arginine demethylases including lysine demethylases (KDM3A, 4A, 4E, 5C) and JmjC domain-containing protein 6 (JMJD6) were included to identify the potential demethylase of p62. Over-expression or knockdown of those arginine demethylases respectively in p62 over-expressed HEK293T cells identified JMJD6 and KDM5C as the potential demethylases of p62 (Figure S6C-D). However, the effect of JMJD6 on p62 was more consistent and significant so that it was selected for further investigation. As expected, knockdown of JMJD6 evidently up-regulated p62 ADMA level, and overexpression of JMJD6 inhibited p62 ADMA activated by PRMT6 (Figure S6E-F). Overall, PRMT6 catalyzes and JMJD6 demethylates p62 ADMA, which could be induced upon ferroptosis induction to enhance the phase separation of p62.

### PRMT6-mediated ADMA increased p62 oligomerization independent of Keap1 interaction upon ferroptosis induction

**Figure 5:**
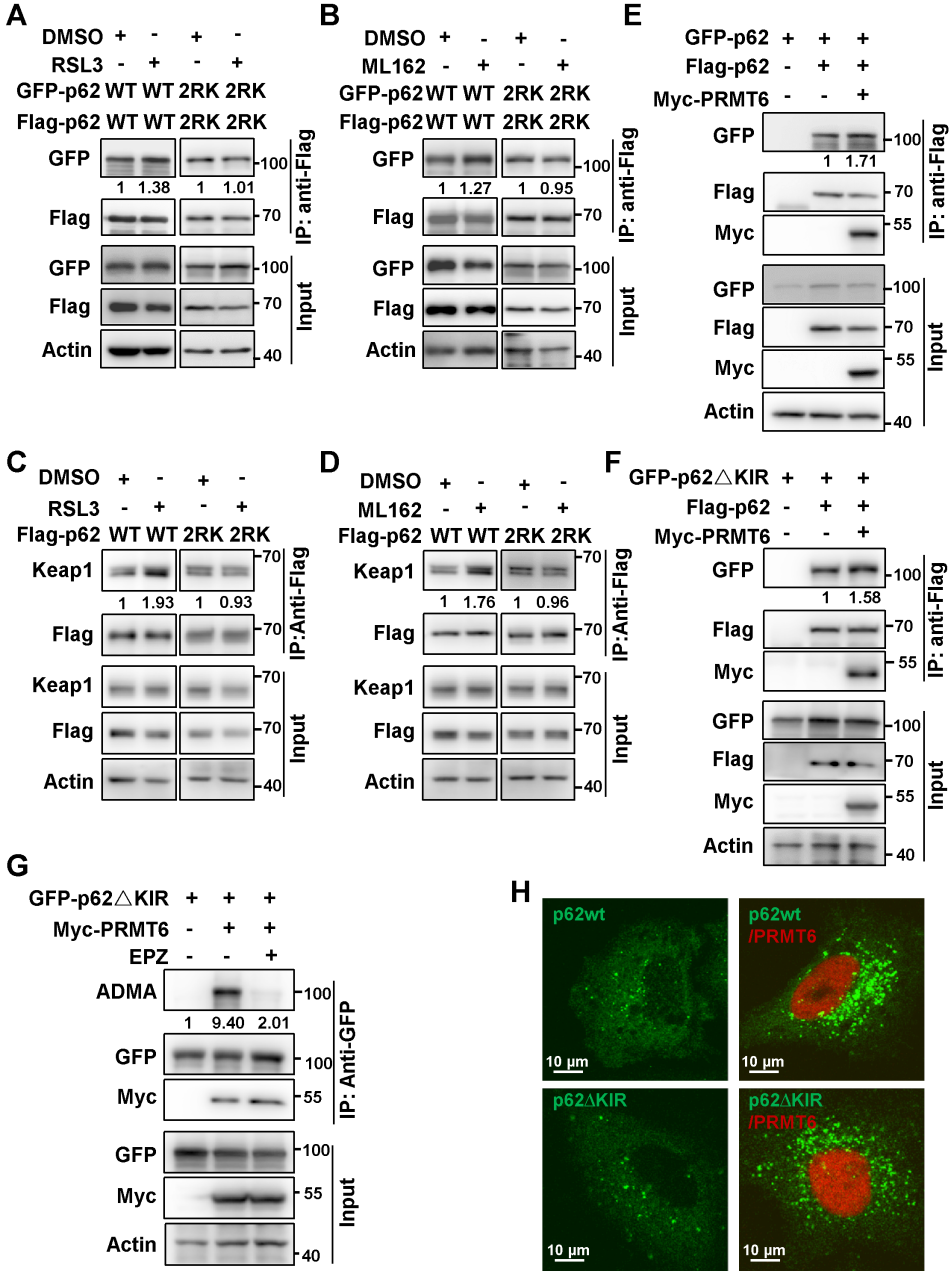
** PRMT6-mediated ADMA increased p62 oligomerization independent of Keap1 interaction upon ferroptosis induction.** A and B. HEK293T cells were co-transfected with Flag-p62-WT and GFP-p62-WT, or Flag-p62-2RK and GFP-p62-2RK, then treated with 3 μM RSL3 (A) or 2 μM ML162 (B) for 12 h, and co-IP was performed with anti-Flag, followed by immunoblotting with GFP and Flag antibodies. And the relative quantified self-interaction of p62-WT or p62-2RK normalized to immunoprecipitated Flag-p62 was shown. C and D. The interaction of Flag-p62-WT or Flag-p62-2RK with Keap1 in Hela cells with Flag-p62-WT or Flag-p62-2RK transfection and 3 μM RSL3 (C) or 2 μM ML162 (D) for 12 h were detected by anti-Flag co-IP and subsequent western blotting. E. HEK293T cells were co-transfected with Flag-p62 and GFP-p62, together with or without Myc-PRMT6, co-IP was performed with anti-Flag, followed by immunoblotting with GFP, Myc and Flag antibodies. F. HEK293T cells were co-transfected with Flag-p62 and GFP tag p62 KIR domain deletion mutant (GFP-p62**△**KIR), together with or without Myc-PRMT6, co-IP was performed with anti-Flag, followed by immunoblotting with GFP, Myc and Flag antibodies. G. GFP-p62**△**KIR and Myc-PRMT6 co-expressed HEK293T cells were incubated with EPZ (EPZ, 10 μM), or DMSO as a control. IP was performed with anti-GFP, and then immunoblotted with ADMA, GFP and Myc antibodies. H. p62-KO cells were transfected with GFP-p62-WT or GFP-p62**△**KIR, together with or without mCherry-PRMT6, then GFP-p62 and mCherry-PRMT6 signal were examined by a confocal microscopy after fixation.

Previously reports discovered that the oligomerization of p62 is indispensable to its phase separation 28, 29. Thus, we further investigated whether PRMT6 mediated ADMA promoted p62 phase separation by increasing its oligomerization. As expected, ferroptosis inducers greatly increased the self-interaction of wild type p62 (p62-WT) but not p62-2RK mutant (Figure 5A-B). Moreover, ferroptosis inducers increased the insoluble fraction of wild type p62, rather than p62-2RK mutant (Figure S7). As a result, ferroptosis inducers significantly promoted the interaction of Keap1 with wild type p62, but not p62-2RK mutant (Figure 5C-D). Accordingly, overexpressed PRMT6 remarkably elevated self-interaction of p62 (Figure 5E). Meanwhile, deletion of KIR domain of p62 (p62∆KIR), which is responsible for Keap1 interaction, didn't affect PRMT6-enhanced p62 self-interaction (Figure 5F). Consistently, PRMT6 still could catalyze the ADMA of p62∆KIR deletion as usual (Figure 5G). And PRMT6 could augment p62 body formation similarly in p62-KO cells expressing GFP-p62∆KIR or GFP-p62-WT (Figure 5H). Thus, we concluded that PRMT6 mediated ADMA increased p62 oligomerization independent of Keap1 interaction during ferroptosis induction.

### PRMT6-mediated p62 ADMA inhibited ferroptosis activation

**Figure 6:**
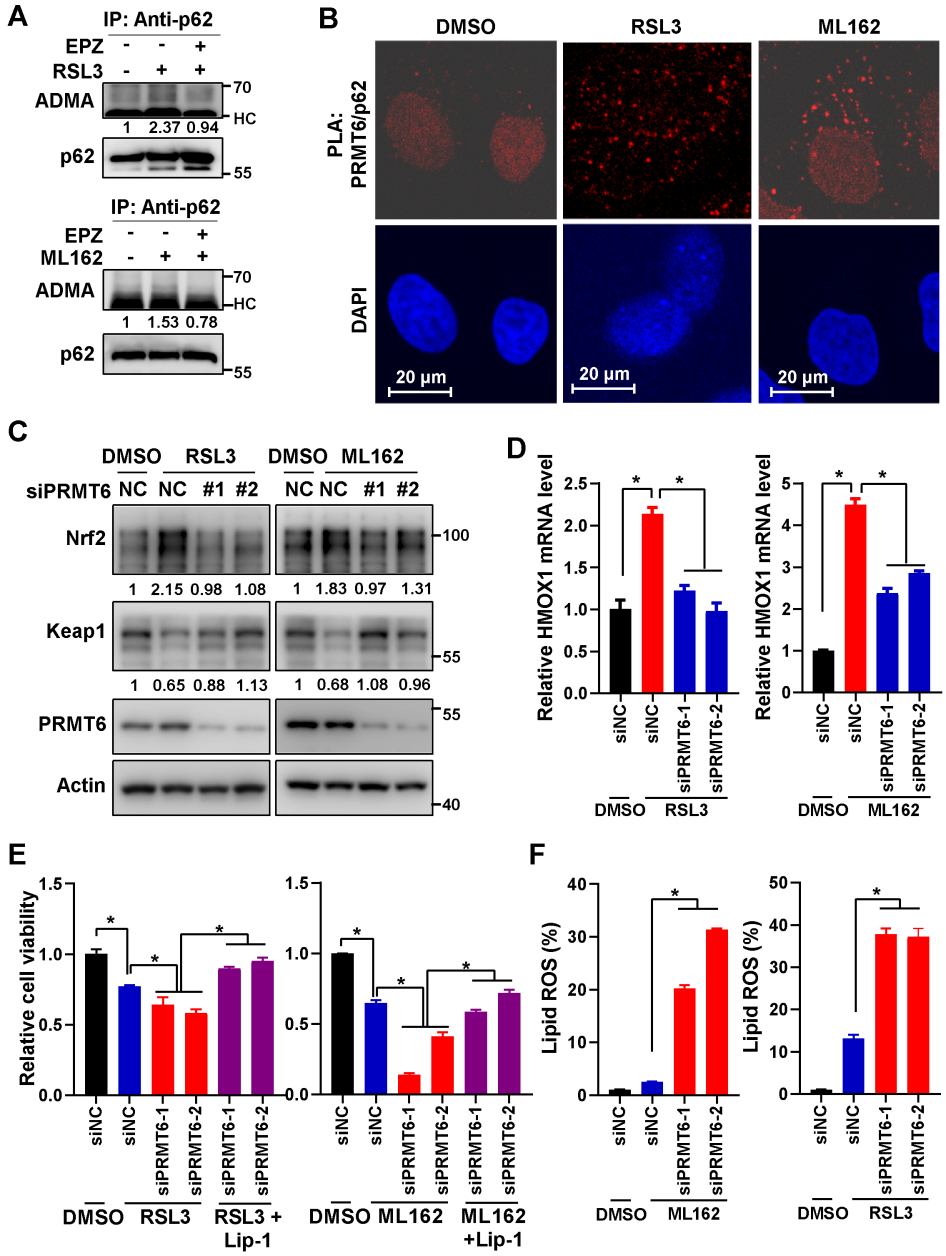
** PRMT6-mediated p62 ADMA inhibited ferroptosis activation.** A. Hela cells were cultured with 3 μM RSL3 (upper) or 2 μM ML162 (down), together with or without EPZ (10μM) for 12 h, then IP was performed with anti-p62, and subsequently blotted with ADMA and p62 antibodies. And relative quantified p62 ADMA normalized to immunoprecipitated p62 was shown. B. After Hela cells treated with 3 μM RSL3 or 2 μM ML162 for 12 h, proximity ligation assay (PLA) was performed with anti-PRMT6 and anti-p62, the nucleus was displayed with DAPI staining, and the photos were captured by a confocal microscopy. The scale bar = 20 μm. C. After PRMT6 knockdown in Hela cells with siRNAs, cells were incubated with 3 μM RSL3 (left) or 2 μM ML162 (right) for 24 h, and western blotting was applied to detected the protein level of Nrf2, Keap1 and PRMT6 with indicated antibodies, actin was used as a loading control. D. After PRMT6 knockdown in Hela cells, cells were incubated with 3 μM RSL3 (left) or 2 μM ML162 (right) for 24 h, and qPCR was adopted to measure the mRNA expression of HMOX1. E. After PRMT6 knockdown in Hela cells, 3 μM RSL3 (left) or 2 μM ML162 (right) were added for 48 h, together with or without Lip-1 (0.25 μM), and cell viability was determined by MTS assay. F. After PRMT6 knockdown in Hela cells, 3 μM RSL3 (left) or 2 μM ML162 (right) were added for 48 h, the lipid ROS was detected with C11-BODIPY reagent.

Next, we confirmed that ferroptosis inducers could induce ADMA of endogenous p62, which could be suppressed by EPZ (Figure 6A). And proximal ligation assay (PLA) revealed that the association of PRMT6 with p62 was increased after ferroptosis induction (Figure 6B). Furthermore, PRMT6 knockdown reversed Nrf2 upregulation and increased Keap1 protein level after ferroptosis induction (Figure 6C and Figure S8A), leading to the downregulation of Nrf2 downstream genes HMOX1 and NQO1 (Figure 6D, Figure S8B-C). As a result, knockdown of PRMT6 increased the cytotoxicity and lipid ROS after ferroptosis induction, which could be rescued by Lip-1 (Figure 6E-F and Figure S8D). However, PRMT6 knockdown failed to do so in p62-KO cells (Figure S8E), confirming that PRMT6 inhibition sensitizes ferroptosis via relevance of p62. To sum up, suppression of p62 ADMA by inhibiting PRMT6 could sensitize ferroptosis via inhibiting Nrf2-dependent anti-ROS response.

### Inhibition of PRMT6-mediated p62 ADMA sensitized ferroptosis in pancreatic cancer

Due to the positive correlation and high expression of p62 and PRMT6 compared to other cancer cell lines (Figure S9A), pancreatic cancer was further selected to verify the role of PRMT6-mediated p62 ADMA in ferroptosis. Actually, ferroptosis inducers did activate endogenous p62 ADMA that would be suppressed by EPZ in pancreatic cancer cells (Figure S9B-C). Similarly, the association of PRMT6 and p62 was augmented in pancreatic cancer cells after ferroptosis induction (Figure S9D). Meanwhile, ferroptosis inducers promoted p62 accumulation and recruited Keap1 into p62 bodies in pancreatic cancer cells (Figure S9E). Thus, knockdown of p62 or PRMT6 after ferroptosis induction reversed Keap1 downregulation and Nrf2 upregulation, as well as the upregulation of Nrf2 downstream genes like NQO1 (Figure S10). As a result, suppression of p62 or PRMT6 increased cytotoxicity and augmented lipid ROS after ferroptosis induction (Figure S11), indicating that inhibition of PRMT6-mediated p62 ADMA sensitized ferroptosis in pancreatic cancer cells.

**Figure 7:**
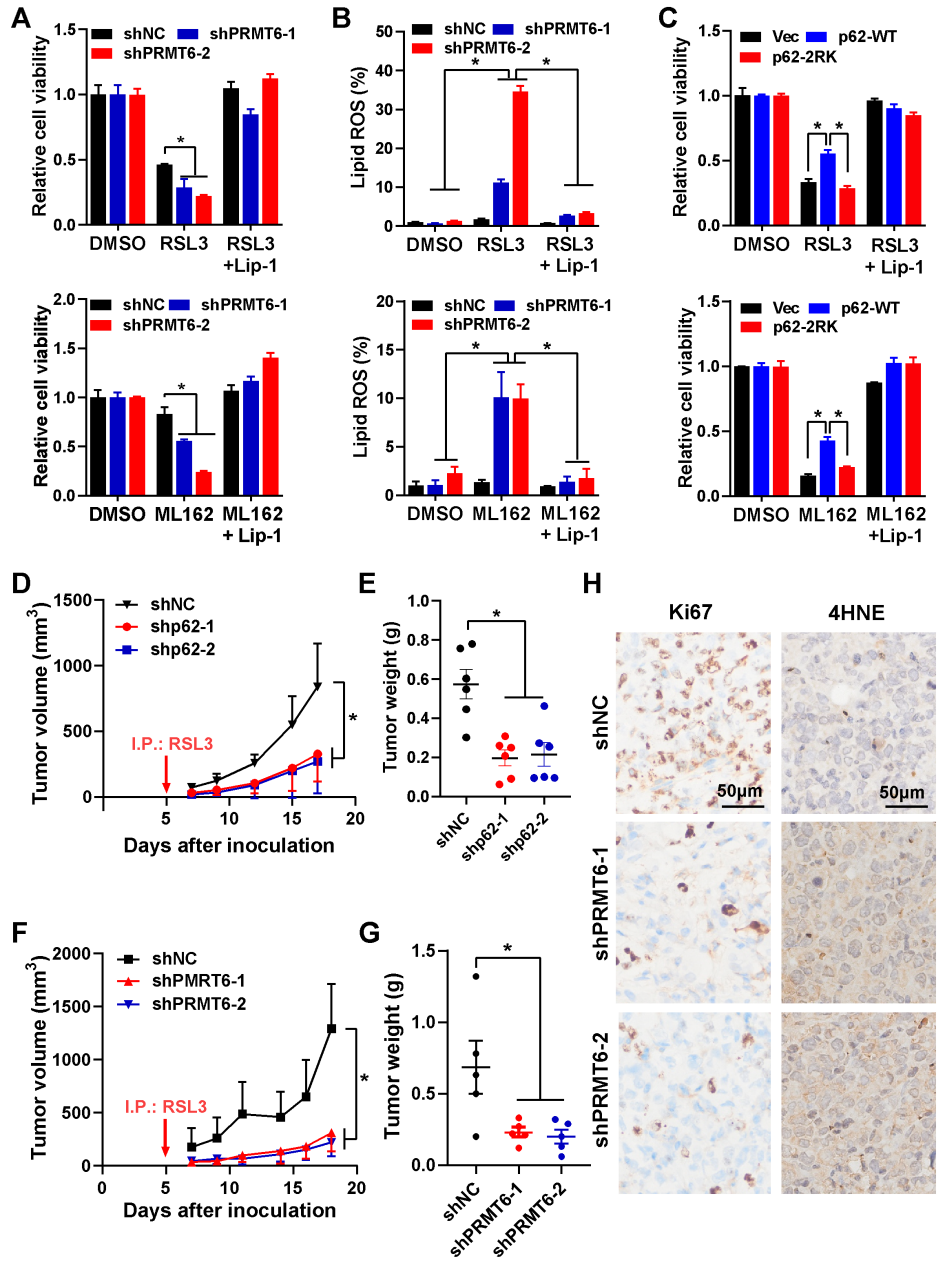
** Inhibition of PRMT6 mediated p62 ADMA sensitized ferroptosis in pancreatic cancer.** A. After MIAPACA-II shNC (MIA-shNC) or shPRMT6 (MIA-shPRMT6) cells were treated with 2 μM RSL3 (upper) or 1 μM ML162 (down) for 48 h, together with or without Lip-1, cell viability was measured by MTS assay. B. After MIA-shNC or MIA-shPRMT6 cells were treated with 2 μM RSL3 (upper) or 1 μM ML162 (down) for 48 h, together with or without Lip-1, the lipid ROS was detected with C11-BODIPY reagent. C. After Flag-p62-WT or 2RK introduced into MIA-shp62 cells, 2 μM RSL3 (upper) or 1 μM ML162 (down) were added for 48 h, together with or without 0.25 μM Lip-1, cell viability was measured by MTS assay. D-E. Xenograft experiment was adopted with subcutaneous inoculation of MIA-shNC or MIA-shp62 cells (n = 6 per group), RSL3 (10 mg/kg) was intraperitoneal injection every other day. Tumor growth curve (D) and tumor weight (E) were shown. F-G. After subcutaneous inoculation of MIA-shNC or MIA-shPRMT6 cells into nude mice (n = 6 per group), RSL3 (10 mg/kg) was intraperitoneal injection every other day. Tumor growth curve (F) and tumor weight (G) were shown. H. MIA-shNC or MIA-shPRMT6 xenograft tumor sections were stained with Ki67 and 4HNE by Immunohistochemistry (IHC).

Finally, MIAPACA-II cells with p62 or PRMT6 stable knockdown were constructed. And the growth of these cells (shp62, shPRMT6 and shNC control) were almost the same as the parental MIAPACA-II cells under normal condition without ferroptosis induction (Figure S12A-C). Just like the transient p62 or PRMT6 knockdown, stably knockdown of p62 or PRMT6 sensitized ferroptosis, which were demonstrated by elevated cell cytotoxicity and augmented lipid ROS after ferroptosis induction (Figure 7A-B and Figure S12D-E). Additionally, reintroduction of p62-WT, but not p62-2RK mutant, could partially attenuate ferroptosis induction in p62 knockdown cells (Figure 7C, Figure S12F-G). Furthermore, tumor xenograft experiment was adopted to evaluate the *in vivo* effect of PRMT6-mediated p62 ADMA. In line with the *in vitro* results, knockdown of p62 or PRMT6 significantly inhibited *in vivo* tumor growth compared to control shNC cells with RSL3 administration (Figure 7D-G and Figure S13A-B). And the protein level of Nrf2, as well as cell proliferation marker Ki-67, were greatly reduced in tumors bearing shp62 or shPRMT6 cells compare to shNC cells. On the contrary, the level of 4NHE, a ferroptosis marker, was remarkably increased in shp62 and shPRMT6 tumors (Figure 7H and Figure S13C-D). Therefore, inhibition of PRMT6-mediated p62 ADMA could significantly sensitize pancreatic cancer cells to ferroptosis both *in vitro* and *in vivo*.

## Discussion

**Figure 8:**
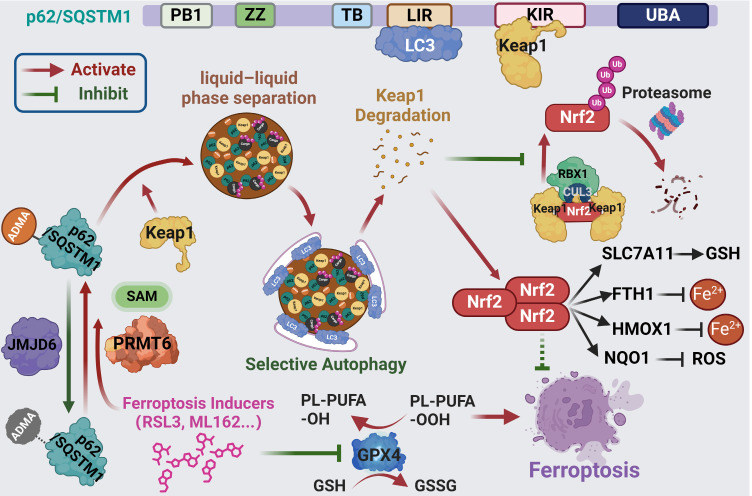
** Scheme of hypothesis.** Ferroptosis inducers including RSL3 and ML162 activates p62 ADMA by increasing its association with PRMT6. And JMJD6 was identified as the potential demethylases of p62 ADMA. The PRMT6 mediated ADMA promotes p62 phase separation to recruit Keap1 into p62 body, which enhances autophagic degradation of Keap1 to upregulate Nrf2 protein level, ultimately transactivating its downstream signaling to confer ferroptosis resistance.

For every action there is an equal and opposite reaction. This is also true for ferroptosis induction which was recently considered as an effectiveness strategy for cancer treatment. However, such equal and opposite reactions will function as the defensive response to attenuate ferroptosis induction, thus compromising the clinical efficacy of ferroptosis inducers in cancer treatment. Recently, p62-Keap1-Nrf2 axis was found to be one of the dominant defense mechanisms contribute to ferroptosis tolerance 13, 14. It would be necessary to abrogate these defensive reactions to maximize the effect of ferroptosis inducers. Unfortunately, how p62 was activated upon ferroptosis induction to protect Nrf2 from keap1-dependent proteasomal degradation remains unknown. Herein, we demonstrated that ferroptosis inducers could promote p62 phase separation via PRMT6-catalyzed ADMA to recruit Keap1 into p62 bodies, which triggers Keap1 autophagic degradation, resulting in the upregulation of Nrf2 protein to compromise ferroptosis activation (Figure 8).

Previously, p62-Keap1-Nrf2 axis was well known to be activated under various oxidative stress to alleviate oxidative damage during various pathophysiology processes including carcinogenesis and drug resistance 30-32. As the important role of p62-Keap1-Nrf2 signaling against oxidative stress, it was not surprisingly that this axis was also activated to mitigate lipid ROS during ferroptosis induction. In addition, Nrf2 could also promote the transcription of several key genes involved in iron metabolism such as the light chain and heavy chain of ferritin (FTL/FTH1), as well as the iron export protein ferroportin (SLC40A1) 8. Furthermore, Nrf2 transactivates a lot of GPX4 related genes including SLC7A11, glutamate cysteine ligase (GCL), glutathione synthetase (GSS) and GPX4 itself 5, 33. Therefore, current findings considered Nrf2 signaling as one of the key decision-makers to neutralize ferroptosis 5. Increasing evidences showed that p62-Keap1-Nrf2 axis was activated during ferroptosis induction in various cancers especially hepatocellular carcinoma (HCC) 13-15. For example, enhanced interaction of p62 and Keap1, p62 phosphorylation were found to prevent Keap1 from targeting Nrf2 for proteasomal degradation, activating Nrf2 downstream signaling and resulting in ferroptosis resistance 13, 14. Therefore, inhibition of p62, Nrf2 or its downstream genes could greatly sensitize ferroptosis, representing a more rational strategy of cancer targeted therapy based on ferroptosis activation. In line with these findings, we confirmed that p62-Keap1-Nrf2 axis was activated by PRMT6-mediated ADMA of p62, and suppression of p62 or Nrf2 could sensitized ferroptosis in pancreatic cancer (Figure 1A-D, Figure S1-2 and Figure S10-11). Targeting PRMT6-mediated ADMA of p62 could be a novel option to synergy with ferroptosis activation in drug discovery and strategy design.

As a multiple functional adaptor protein, p62 is well known to be involved in mediating selective autophagy, which also works as a central hub to activate several oncogenic signaling such as Nrf2 and mTORC1 signaling 34. Therefore, p62 is found to be an oncogenic protein that is frequently up-regulated in various cancers.

p62 body is a non-membrane compartment condensed by liquid-liquid phase separation of p62, which would gather ubiquitinated cargoes as well as Keap1 and enable them for autophagic degradation, thus playing prominent roles in the regulation of p62 associated functions 23, 26. Recent reports supported that p62 phase separation stimulated the recruitment of Keap1 into p62 body to enhance Nrf2 dependent detoxification under oxidative stress 23. Interestingly, we demonstrated in this study that ferroptosis inducers engaged p62 phase separation to recruit Keap1 into p62 body, thus activating p62-Keap1-Nrf2 axis to ameliorate ferroptosis (Figure 1 and Figure S3).

Post-translational modification (PTM) and protein-protein interaction play important roles in the phase separation of various proteins. It was reported that interacting partners such as DAXX promoted p62 phase separation to enrich Keap1 into p62 body 28, 35. On the other hands, either S403, S405, S409 phosphorylation, or lysine 420 (K420), K435 acetylation in the UBA domain enhance p62 phase separation by increasing its binding affinity to ubiquitinated proteins 27, 36. On the contrary, K7 ubiquitination at the PB1 domain indispensable for p62 oligomerization, or K420 ubiquitination would reduce p62 body formation by decreasing its oligomerization or the binding affinity to ubiquitinated proteins, respectively 37, 38. It seems that the oligomerization and the binding affinity of the ubiquitinated cargoes of p62 play a leading role in its phase separation. And in our study, we identified that PRMT6-mediated ADMA at R183 and R217 could promote its phase separation by augmenting p62 oligomerization (Figure 2-5). Additionally, lysine demethylases JMJD6 were identified to be the potential demethylases of p62. Certainly, there could be other enzymes important to the ADMA of p62 in addition to PRMT6 and JMJD6. For example, over-expression or knockdown of KDM5C could also affect p62 ADMA (Figure S6C-D).

Ferroptosis inducers activated PRMT6-mediated p62 ADMA to recruit Keap1 into p62 body, which attenuated ferroptosis by upregulating Nrf2 signaling. Reintroducing wild type p62, but not p62-2RK mutant, could partially mitigate ferroptosis in p62-KO cells (Figure 2 and 6). Though we found that PRMT6 mainly localized in the nucleus, and only a small fraction of p62 translocated in the nucleus, we indeed observed the interaction of PRMT6 and p62, which would further be elevated by ferroptosis inducers treatment (Figure 3 and 5). In addition, Proximal ligation assay (PLA) indicated the association of PRMT6 and p62 occurred in both cytoplasm and nucleus upon ferroptosis induction (Figure 6), which supported that a small amount of PRMT6 might translocate to the cytoplasm. Besides, it was reported that p62 is a high frequent nucleo-cytoplasmic shuttling protein 39. And protein nuclear exporting inhibitor treatment did largely increase the nuclear location of p62 (data not shown). Therefore, some PRMT6 might catalyze p62 ADMA after translocating to the cytoplasm. On the other hand, p62 might be methylated by PRMT6 in the nucleus, and then exported into the cytoplasm. Nevertheless, it would be interesting to explore how PRMT6-p62 interaction was regulated upon ferroptosis induction. While the expression of PRMT6 was not changed after ferroptosis, we propose that either p62 or PRMT6 was post-translationally modified to promote their interaction upon ferroptosis activation.

Finally, both PRMT6 and p62 are highly expressed in pancreatic cancer cells, resulting in the elevated PRMT6-mediated p62 AMDA, which greatly attenuates the efficiency of ferroptosis induction. As a result, suppression of PRMT6 or p62 remarkably sensitized ferroptosis in pancreatic cancer both *in vitro* and *in vivo* (Figure 7 and Figure S9-13). Therefore, targeting PRMT6-mediated p62 ADMA should be considered in cancer treatments resulting in ferroptosis activation in pancreatic cancer, and probably other cancers. We believe that this finding would be important to improve the drug discovery and trial design for new anti-cancer drugs like PRMT6 inhibitor and other chemotherapeutics activating ferroptosis.

In conclusion, PRMT6-dependent p62 ADMA promotes its oligomerization and phase separation to recruiting Keap1 into p62 bodies, thus preventing Nrf2 from proteasomal degradation to confer ferroptosis tolerance. Therefore, targeting PRMT6-mediated p62 ADMA might be a potential synergistic strategy for ferroptosis-inducing therapies in pancreatic cancer, and probably other cancers.

## Supplementary Material

Supplementary figures and table.
