# Supplementary material for: PRMT6-mediated ADMA promotes p62 phase separation to form a negative feedback loop in ferroptosis
Source: Theranostics. 2024 Jul 2;14(10):4090–106. doi: 10.7150/thno.94789 (PMC11234273; doi:10.7150/thno.94789)
Supplement: Supplementary file 1 — Supplementary figures and table. [file thnov14p4090s1.pdf]

Supplementary Figures

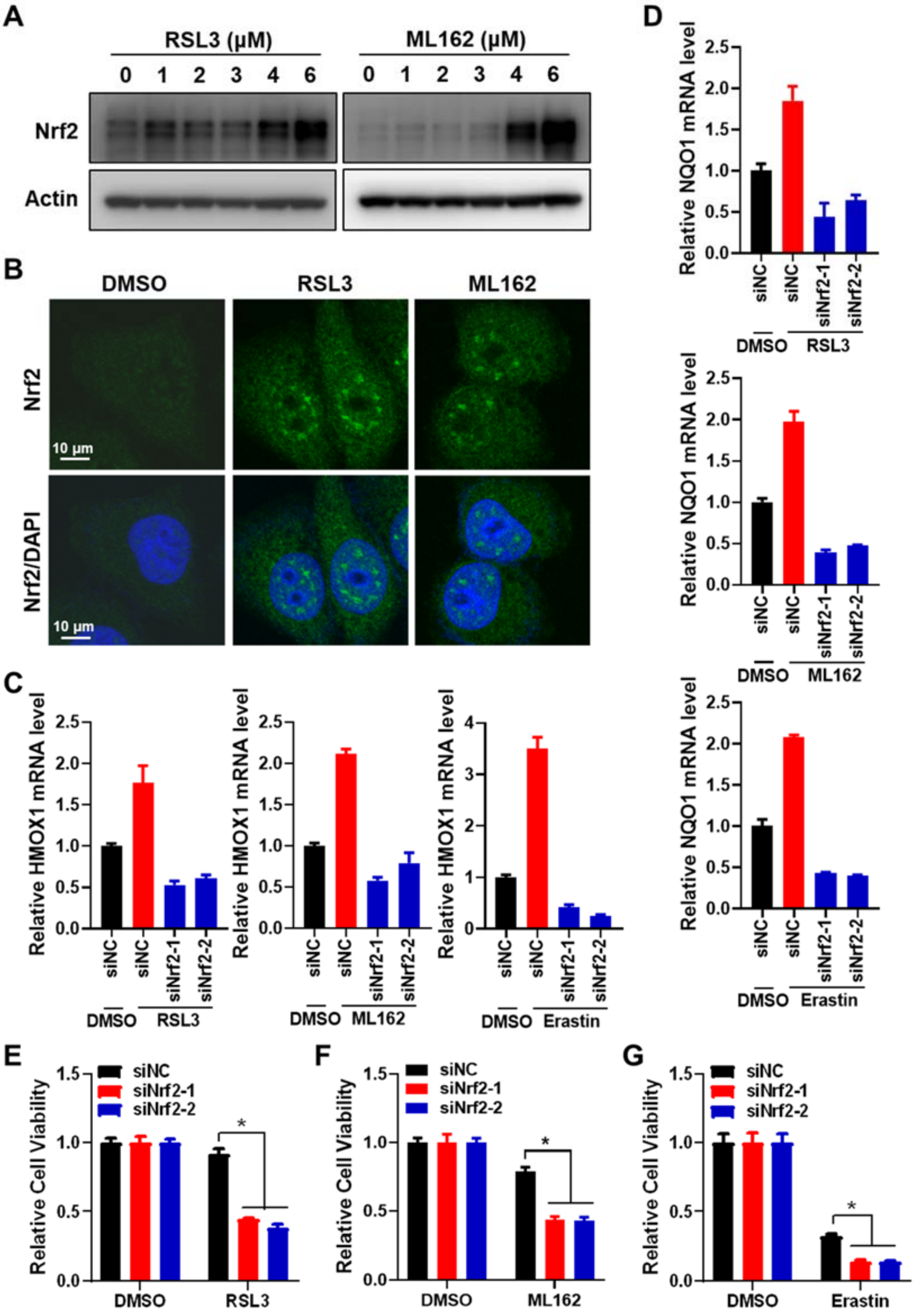

Figure S1. Nrf2 was upregulated to compromise ferroptosis

A. Nrf2 protein level with dose-dependently RSL3 or ML162 24 h treatment in Hela cells was determined by western blotting.

B. Nrf2 protein level and distribution in Hela cells with DMSO, RSL3 or ML162 24 h treatment was detected with immunofluorescence (IF).

C and D. The mRNA expression of HMOX1 (B) or NQO1 (C) in Hela cells with Nrf2 knockdown and RSL3 (3 μM), ML162 (2 μM) or Erastin (8 μM) treatment for 24 h respectively was detected by qPCR.

E-G. After Nrf2 knockdown with siRNA transient transfection, Hela cells were incubated with 3 μM RSL3 (B), 2 μM ML162 (C), 8 μM Erastin (D) for 48 h respectively, and cell viability was measured with MTS assay.

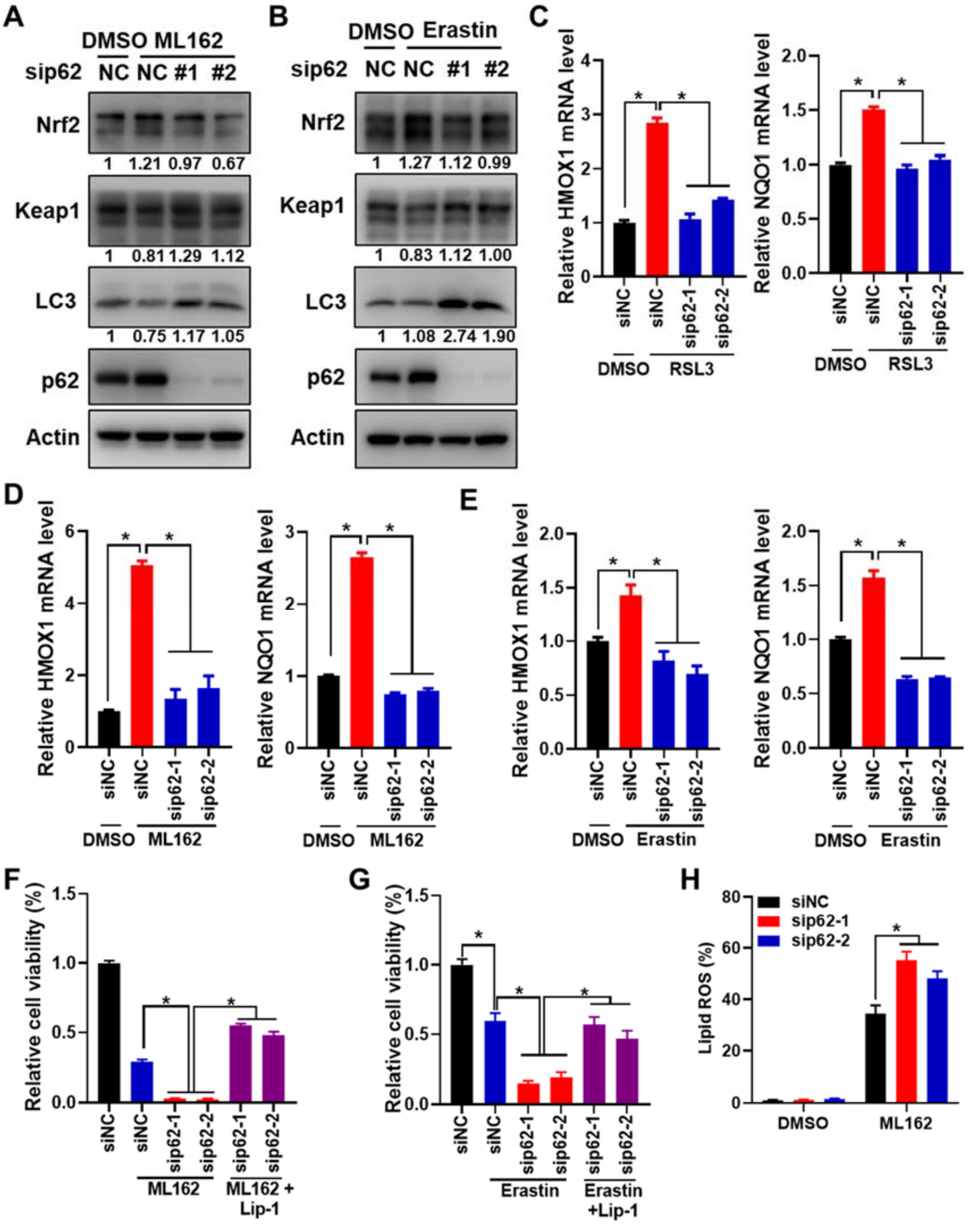

Figure S2. p62 is important to Nrf2 upregulation during ferroptosis activation

A-B. The protein level of Nrf2, Keap1 and p62 in Hela cells with p62 knockdown and 2 μM ML162 (A) or 8 μM Erastin (B) treatment for 24 h, were determined by western blotting. Actin was used as a loading control. And relative quantified protein level of Nrf2, Keap1 and LC3-II normalized to Actin were shown below the corresponding band.

C-E. HMOX1 (left) and NQO1 (right) mRNA expression in Hela cells with p62 knockdown and 3 μM RSL3 (C), 2 μM ML162 (D) or 8 μM Erastin (E) incubation for 24 h respectively, were detected by qPCR.

F-G. After p62 knockdown, Hela cells were dealt with ML162 (F) or Erastin (G) for 48 h respectively, together with or without liproxstatin 1 (Lip-1, 0.25 μM), and cell viability was measured with MTS assay.

H. After p62 knockdown, Hela cells were treated with 2 μM ML162 for 48 h, lipid ROS was detected with C11-BODIPY reagent by flow cytometer.

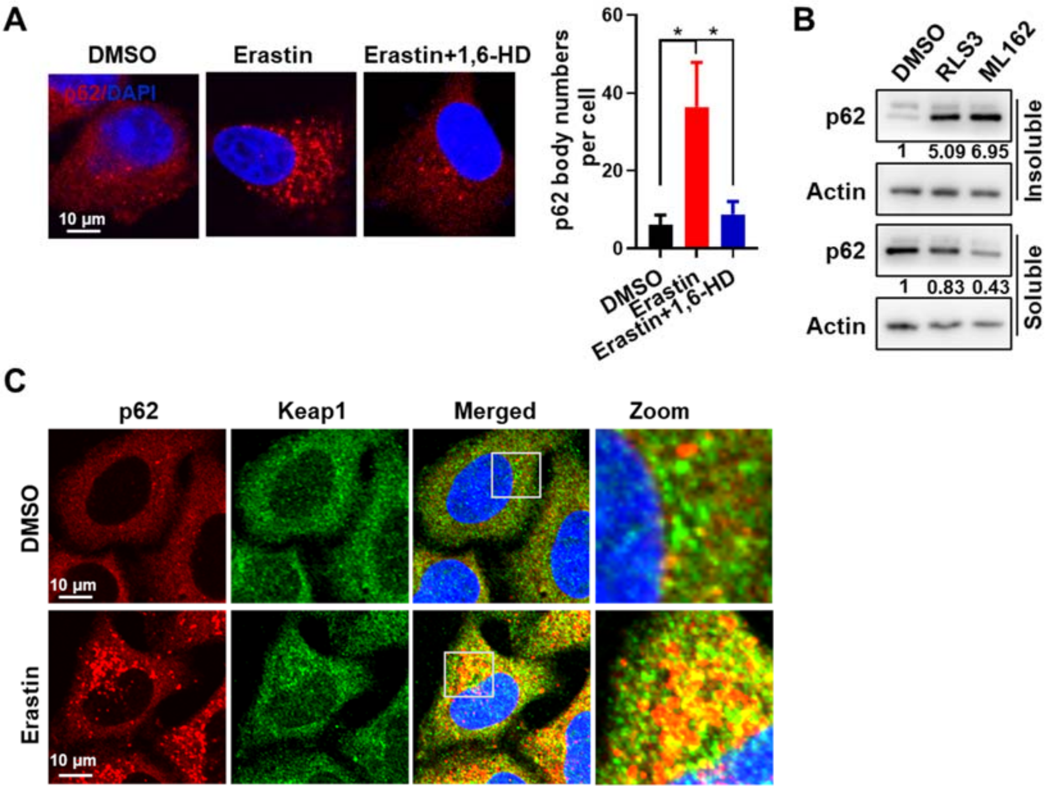

Figure S3. Ferroptosis inducers promoted p62 phase separation to recruit Keap1 into p62 body

A. p62 distribution in Hela cells cultured with 8 μM Erastin for 12 h, were fixed and treated with or without 5% 1,6-Hexanediol (1,6-HD), was determined by immunofluorescence (IF). And the numbers of p62 body per cell (n = 5) were counted and shown and mean ± SD.

B. Soluble-insoluble fractions of p62 after RSL3 or ML162 24 h incubation in

Hela cells were determined by western blotting. And relative quantified protein level of p62 normalized to Actin in soluble and insoluble fractions were shown. C. Co-localization of p62 and Keap1 in Hela cells with Erastin (8 μM) 12 h incubation was detected by IF.

The scale bar is 10 μm.

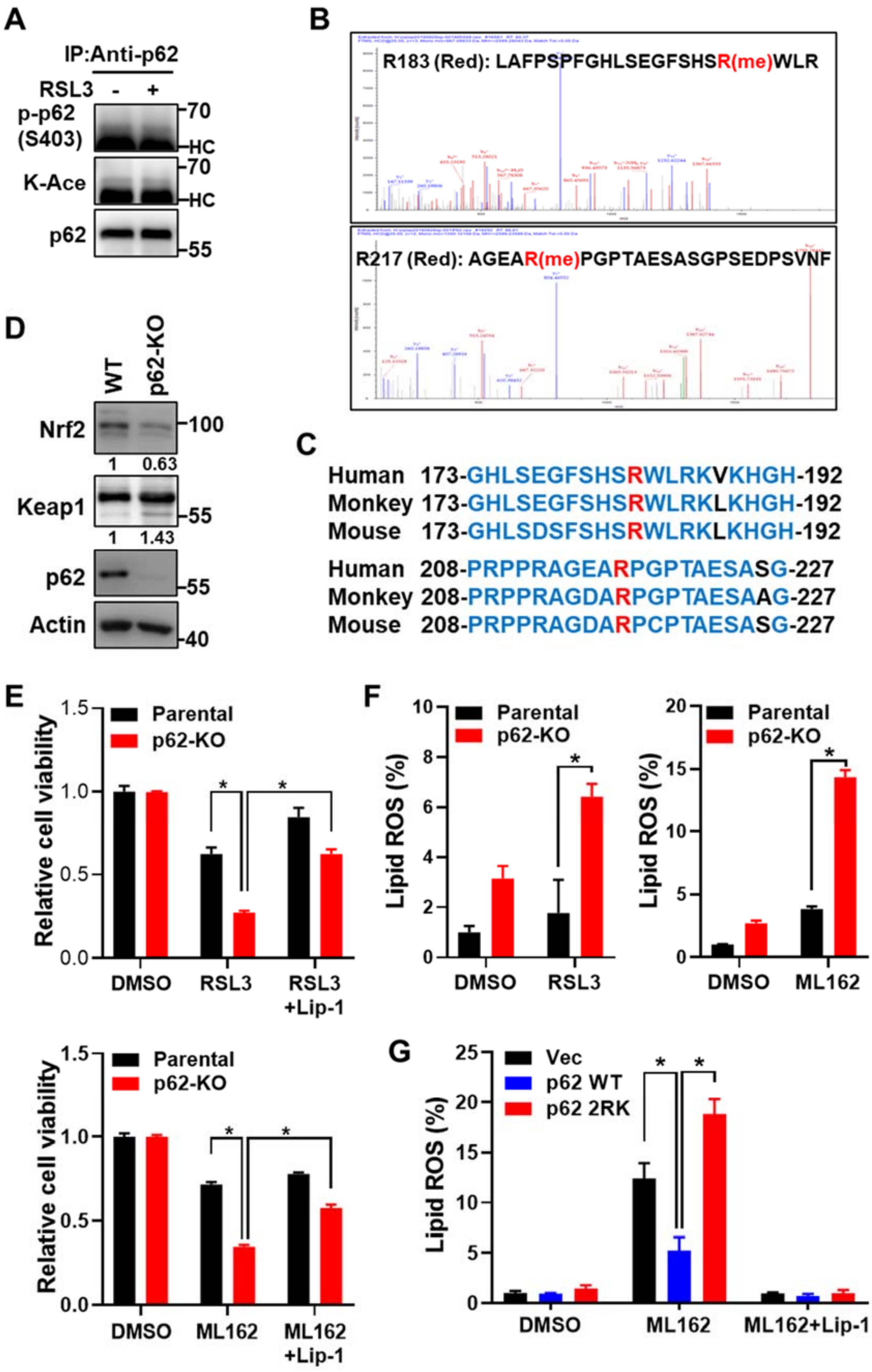

Figure S4. Ferroptosis inducers activated p62 R183/R217 ADMA

A. The lysates of Hela cells incubated with RSL3 (3 μM) or DMSO for 12 h were

immunoprecipitated with anti-p62, then immunoblotted with p62 ser403 phosphorylation (p-p62(S403)), pan lysine acetylation (K-ace) and p62 antibodies.

B. The represented p62 arginine 183 or 217 (R183 or R217) methylation mass spectrum (MS) analysis data was shown.

C. The conserved R183 or R217 amino acid sequences in human, monkey and mouse were shown.

D. The expression of p62, Nrf2 and Keap1 in p62-KO or parental Hela cells were detected by western blotting with indicated antibodies.

E. Cell viability of Hela or p62-KO cells with 2 μM RSL3 (upper) or 1 μM ML162 (down) 48 h incubation, together with Lip-1 (0.25 μM), was determined by MTS assay.

F. The lipid ROS of Hela or p62-KO cells with RSL3 (left) or ML162 (right) 48 h incubation was detected with C11-BODIPY reagent.

G. The lipid ROS of p62-KO cells with Flag-p62-WT, 2RK or control vector (Vec) overexpression, and ML162 (1 μM) 48 h treatment, was detected with C11-BODIPY reagent.

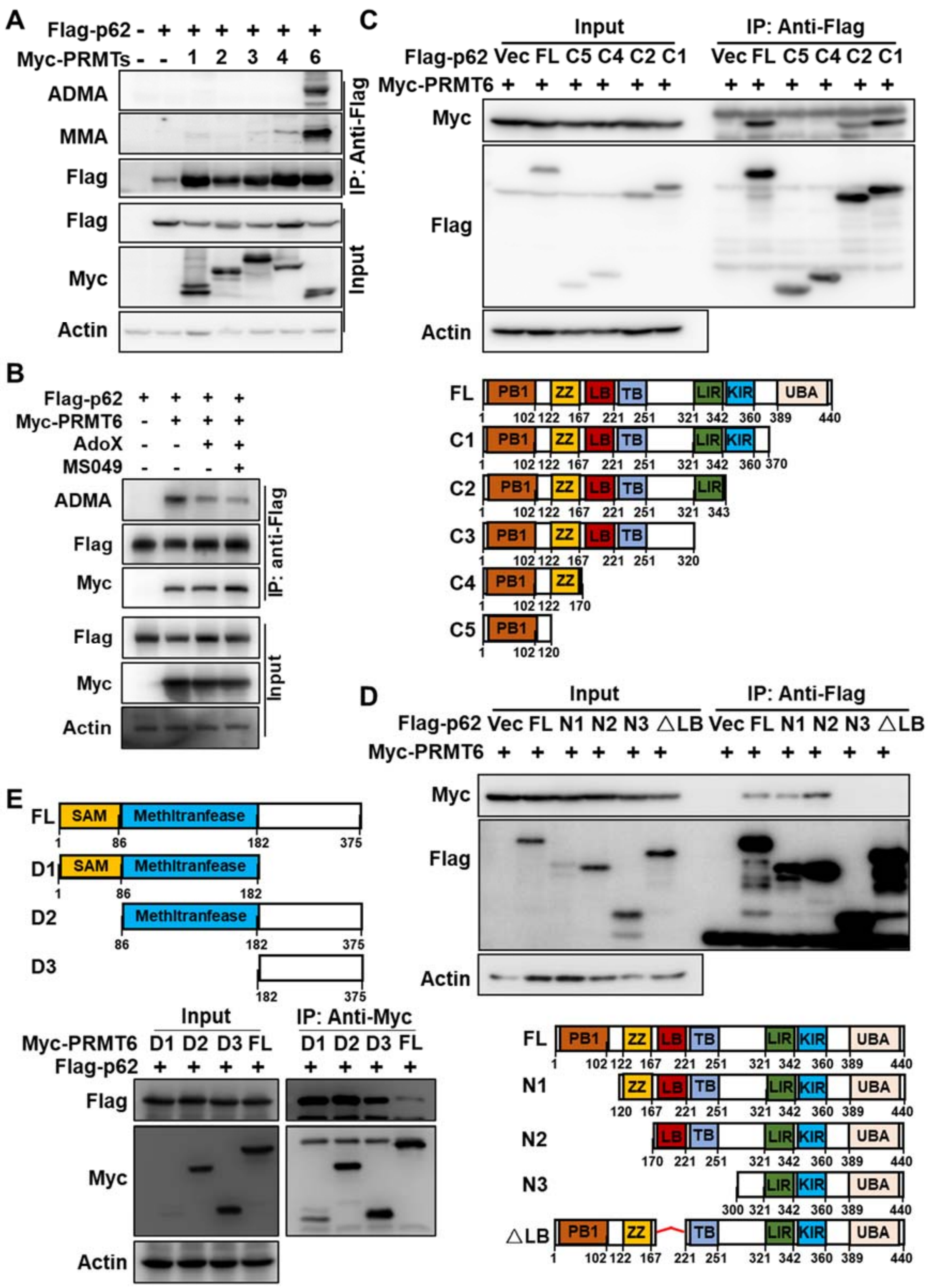

Figure S5. PRMT6 mediated p62 ADMA by interacting with the LB domain of p62

A. Flag-p62 was co-transfected with different PRMTs (Myc-PRMT1, 2, 3, 4, 6) into HEK293T cells respectively, and IP was performed with anti-Flag, subsequently immunoblotting with ADMA, MMA and Flag antibodies.

B. HEK293T cells were co-transfected with Flag-p62 and Myc-PRMT6, then overnight treated with AdoX (5 μM) or MS049 (10 μM), and IP was performed with anti-Flag, followed by immunoblotting with ADMA, Myc and Flag antibodies.

C. HEK293T cells were co-transfected with Flag-p62 or its C terminal truncations (deleted from C terminal as shown) and Myc-PRMT6, and co-IP was performed with anti-Flag, then immunoblotting with Myc and Flag antibodies.

D. HEK293T cells were co-transfected with Flag-p62, its N terminal truncations (deleted from N terminal as shown) or LB domain deletion (△LB) mutant and Myc-PRMT6, and co-IP was performed with anti-Flag, then immunoblotting with Myc and Flag antibodies.

E. HEK293T cells were co-transfected with Flag-p62 and Myc-PRMT6 or its deletions as shown, and co-IP was performed with anti-Myc, then immunoblotting with Myc and Flag antibodies.

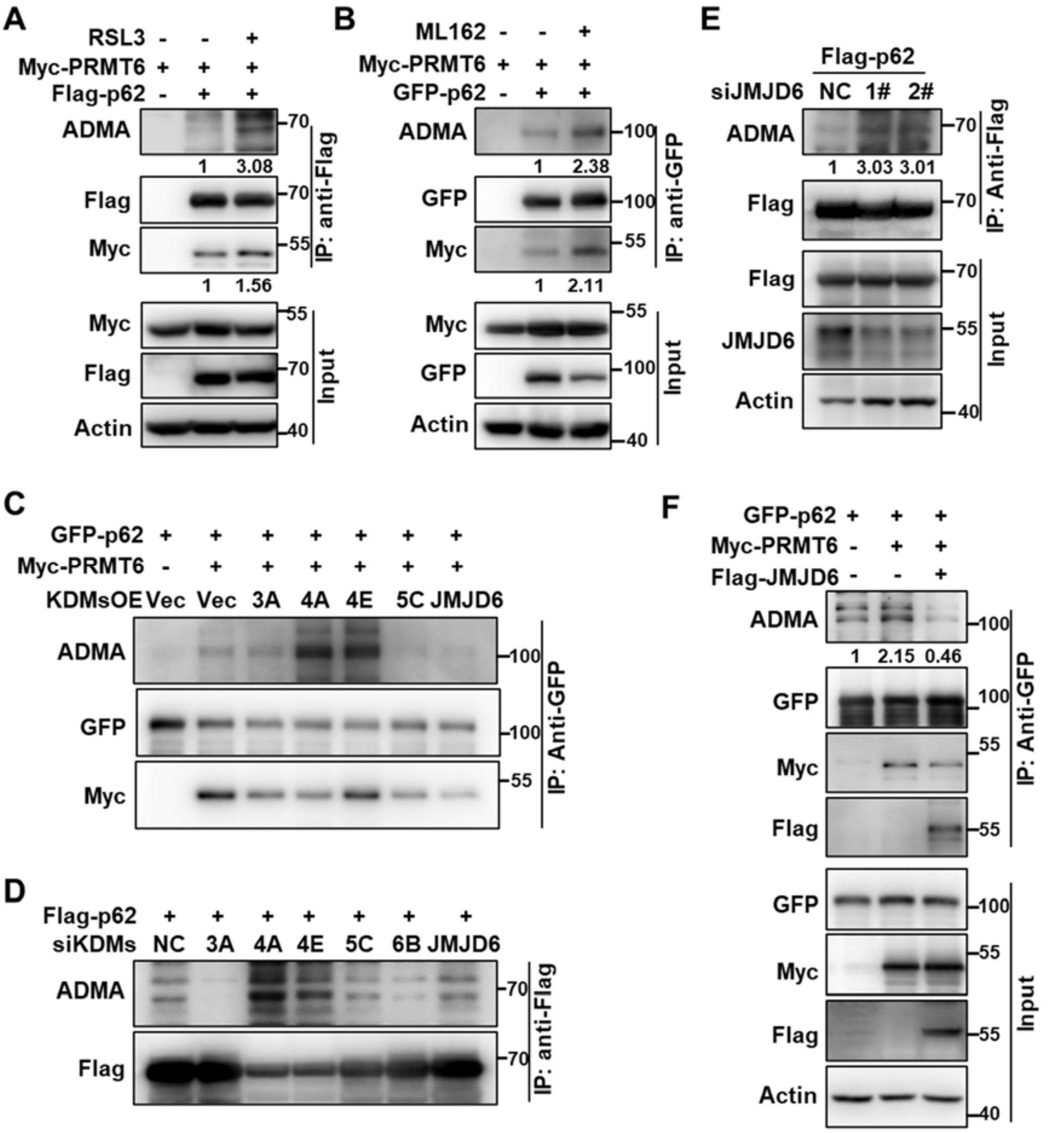

Figure S6. JMJD6 is the arginine demethylase of p62

A-B. HEK293T cells were co-transfected with exo-p62 and Myc-PRMT6, then treated with RSL3 (3 μM) or ML162 (2 μM) for 12 h. After co-IP, immunoblotting was performed with ADMA, Myc and Flag antibodies. And relative quantified p62 ADMA level and Myc-PRMT6 binding level normalized to the immunoprecipitated Flag-p62 were shown below the corresponding band.

C. GFP-p62 was co-transfected with Myc-PRMT6 and different Flag tagged arginine demethylases (KDM3A, KDM4A, KDM4C, KDM5C or JMJD6) respectively into HEK293T cells. And co-IP was performed with anti-GFP, followed by immunoblotting with ADMA, GFP and Myc antibodies.

D. HEK293T cells were co-transfected with Flag-p62 and siRNAs of arginine demethylases (siKDM3A, siKDM4A, siKDM4C, siKDM5C, siKDM6B or JMJD6) respectively. And IP was performed with anti-Flag, followed by immunoblotting with ADMA and Flag antibodies.

E. HEK293T cells were co-transfected with Flag-p62 and siJMJD6s, IP was performed with anti-Flag, followed by immunoblotting with ADMA, JMJD6 and Flag antibodies. And relative quantified p62 ADMA level normalized to the immunoprecipitated Flag-p62 were shown.

F. GFP-p62 was co-transfected with Myc-PRMT6 and Flag-JMJD6 into HEK293T cells. And co-IP was performed with anti-GFP, followed by immunoblotting with ADMA, GFP and Myc antibodies.

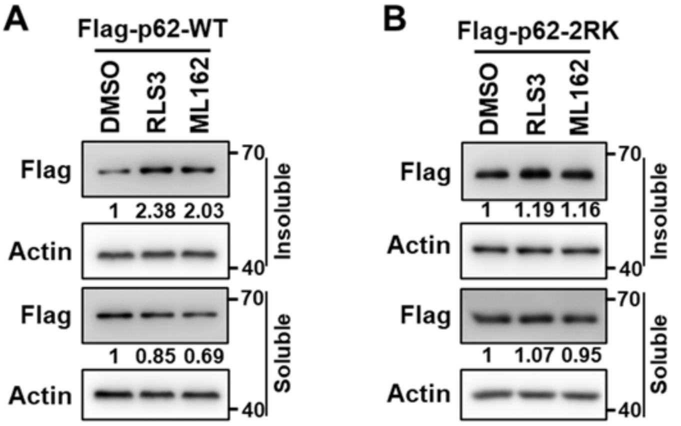

Figure S7. Ferroptosis inducers increased the oligomerization of p62 dependent on R183 and R217 methylation

A and B. Soluble-insoluble fractions of Flag-p62-WT (A) or Flag-p62-2RK (B) mutant after RSL3 or ML162 12 h incubation in Hela cells with Flag-p62-WT or Flag-p62-2RK transient transfection were determined by western blotting. And relative quantified protein level of Flag-p62 normalized to Actin in soluble and insoluble fractions were shown.

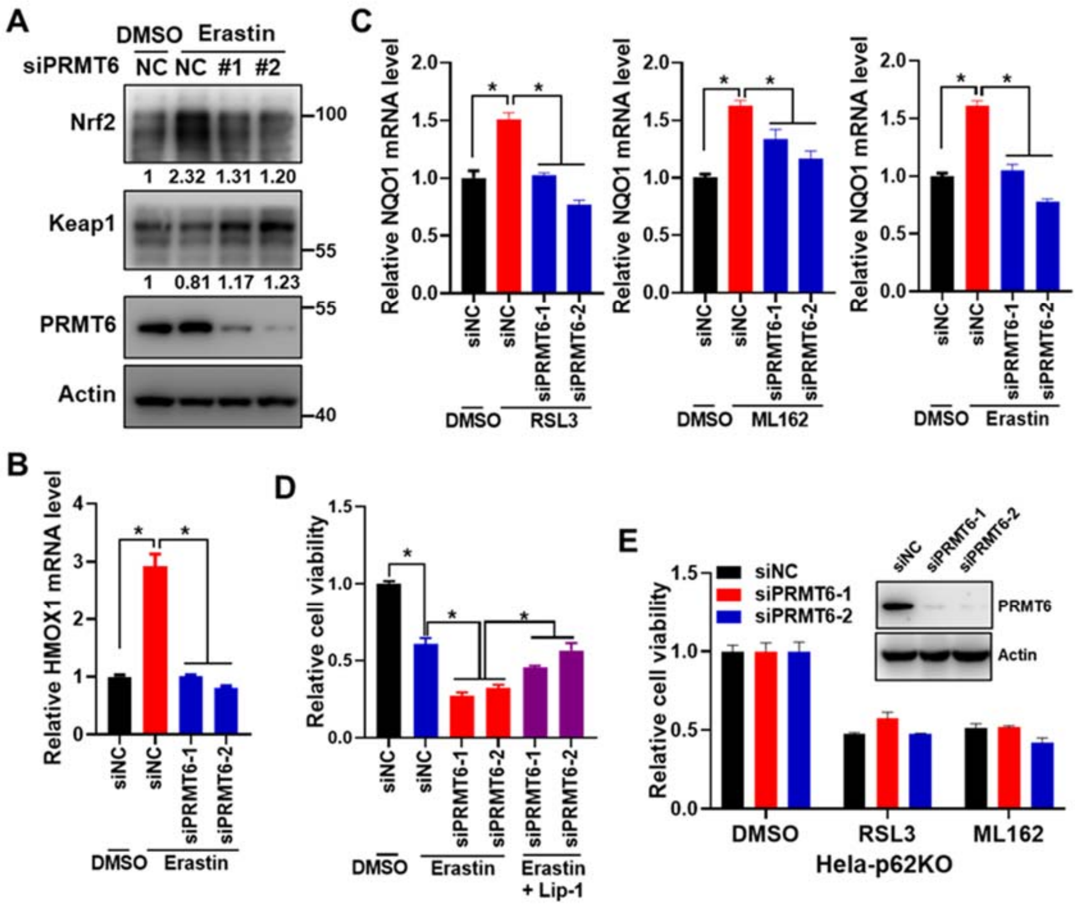

Figure S8. Activation of PRMT6-mediated p62 ADMA resulted in ferroptosis resistance

A. PRMT6 knockdown Hela cells were incubated with 8 μM Erastin for 24 h, and western blotting was applied to detected the protein level of Nrf2, Keap1 and PRMT6, actin was used as a loading control. And relative quantified protein level of Nrf2 and Keap1 normalized to Actin were shown.

B. PRMT6 knockdown Hela cells were incubated with Erastin for 24 h, qPCR was adopted to measure the mRNA expression of HMOX1.

C. PRMT6 knockdown Hela cells were incubated with RSL3, ML162 or Erastin for 24 h, qPCR was performed to measure the mRNA expression of NQO1.

D. PRMT6 knockdown Hela cells were incubated with Erastin for 48 h, together with or without Lip-1, cell viability was determined by MTS assay.

E. p62-KO cells were transfected with PRMT6 siRNAs, then incubated with 1 μM ML162 for 48 h, and cell viability was determined by MTS assay. PRMT6 knockdown was validated by immunoblotting.

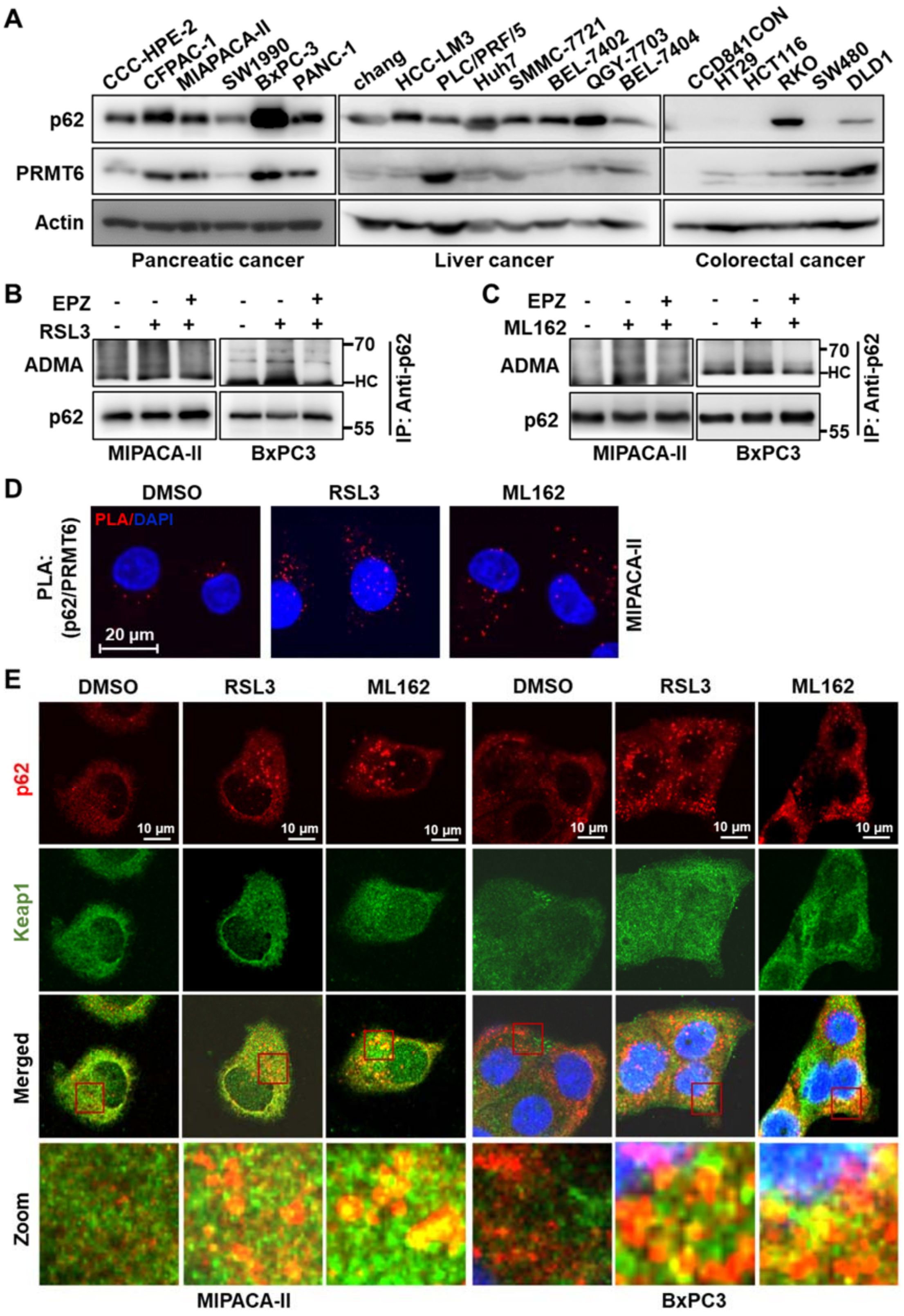

Figure S9. Ferroptosis inducers augmented PRMT6-dependent ADMA to promote p62 body formation in pancreatic cancer cells

A. Protein level of p62 and PRMT6 were determined by western blotting in

pancreatic, liver and colorectal cancer cell lines, actin was used as a loading control.

B-C. MIAPACA-II or BxPC3 cells were treated with 3 μM RSL3 (B) or 2 μM ML162 (C) for 12 h, together with or without 10 μM EPZ, IP was performed with anti-p62, followed by immunoblotting with ADMA and p62 antibodies.

D. After MIAPACA-II cells treated with 3 μM RSL3 or 2 μM ML162 for 12 h, PLA was performed with anti-p62 and anti-PRMT6, the nucleus was displayed with DAPI staining, and the photos were captured by a confocal microscopy. The scale bar = 20 μm.

E. MIAPACA-II or BxPC3 cells were treated with 3 μM RSL3 or 2 μM ML162 for 12 h, IF was performed with anti-p62 and anti-Keap1, and photos were captured by a confocal microscopy. The scale bar = 10 μm.

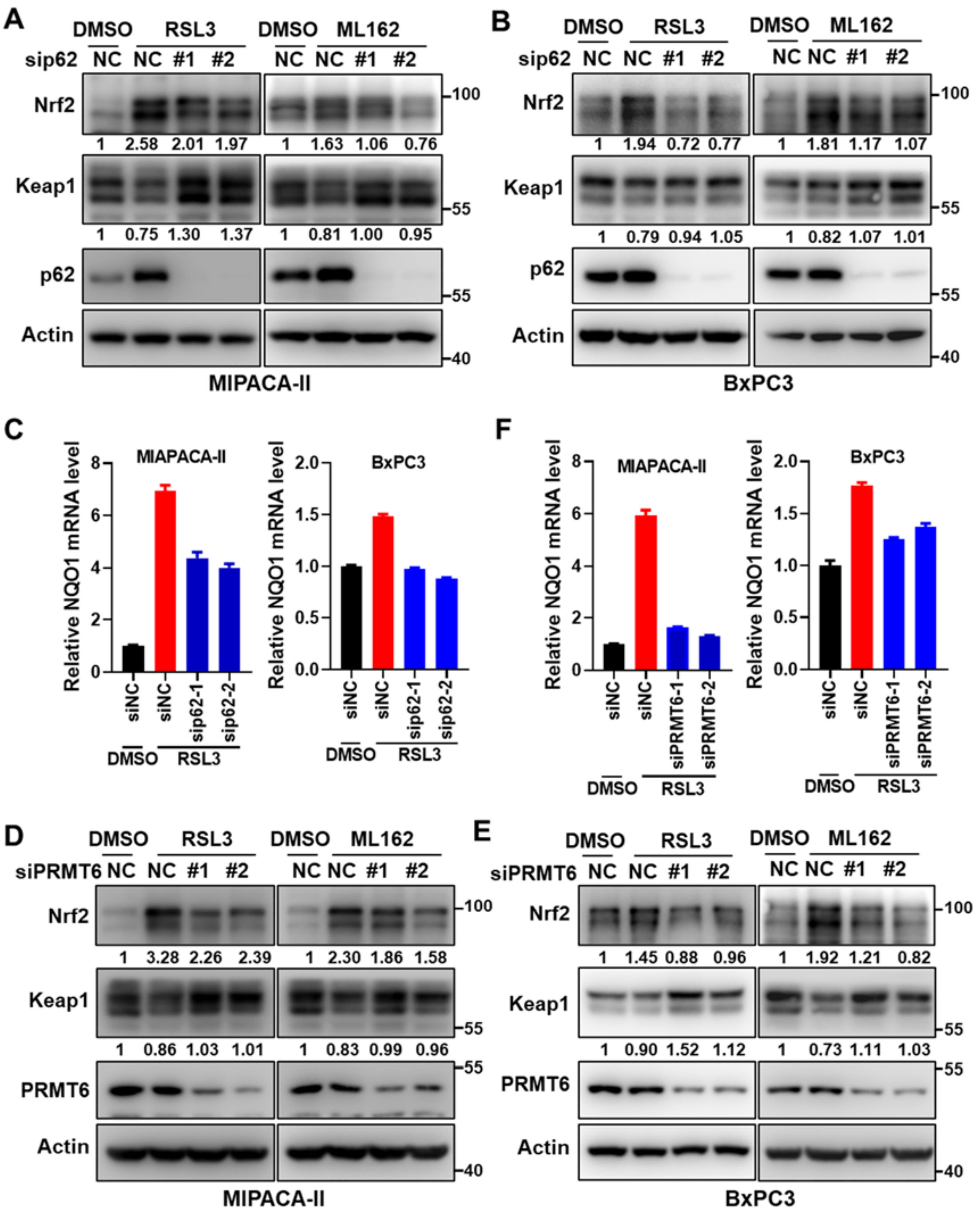

Figure S10. Ferroptosis inducers activated p62-Keap1-Nrf2 axis in pancreatic cancer cells

A-B. After MIAPACA-II or BxPC3 cells were transfected with p62 siRNAs, cells were then incubated with 3 μM RSL3 (A) or 2 μM ML162 (B) for 24 h, western blotting was applied to detect the protein level of Nrf2, Keap1 and p62, actin was used as a loading control. And relative quantified protein level of Nrf2 and Keap1 normalized to Actin were shown.

C. After p62 knockdown in MIAPACA-II or BxPC3 cells, cells were incubated with 3 μM RSL3 for 24 h, qPCR was adopted to measure the mRNA expression of NQO1.

D-E. After MIAPACA-II or BxPC3 cells were transfected with PRMT6 siRNAs, cells were then incubated with 3 μM RSL3 (A) or 2 μM ML162 (B) for 24 h, western blotting was applied to detect the protein level of Nrf2, Keap1 and PRMT6. And relative quantified protein level of Nrf2 and Keap1 normalized to Actin were shown.

F. After PRMT6 knockdown in MIAPACA-II or BxPC3 cells, cells were incubated with RSL3 for 24 h, qPCR was adopted to measure the mRNA expression of NQO1.

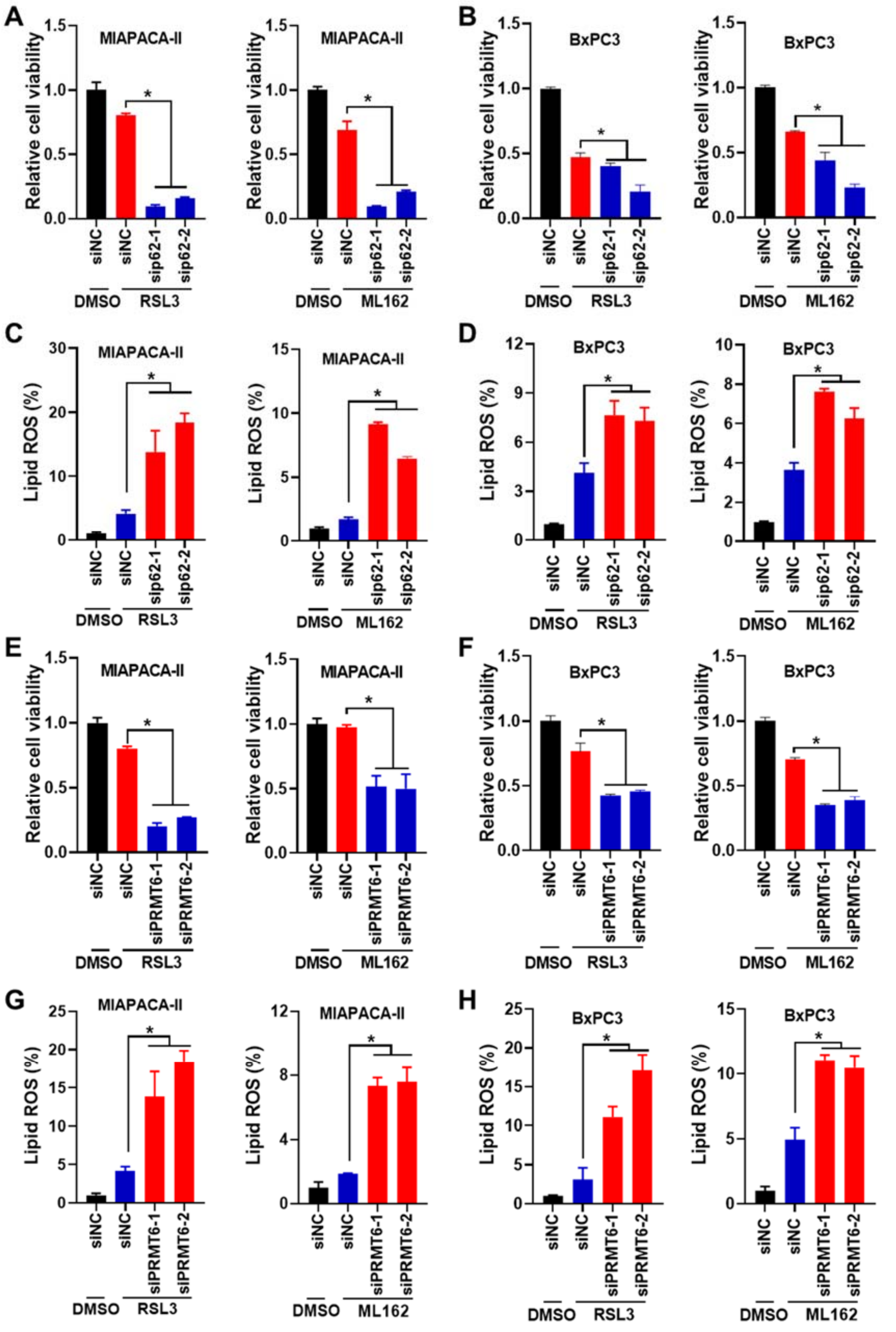

Figure S11. Suppression of p62 or PRMT6 sensitized ferroptosis in pancreatic cancer cells *in vitro*

A-B. After p62 knockdown in MIAPACA or BxPC3 cells, 3 μM RSL3 or 2 μM

ML162 was added for another 48 h, cell viability was measured by MTS assay. C-D. After p62 knockdown in MIAPACA or BxPC3 cells, RSL3 or ML162 was added for another 48 h, the lipid ROS was measured with C11-BODIPY reagent. E-F. After PRMT6 knockdown in MIAPACA or BxPC3 cells, 3 μM RSL3 or 2 μM ML162 was added for another 48 h, cell viability was measured by MTS assay. G-H. After PRMT6 knockdown in MIAPACA or BxPC3 cells, RSL3 or ML162 was added for 48 h, the lipid ROS was measured with C11-BODIPY reagent.

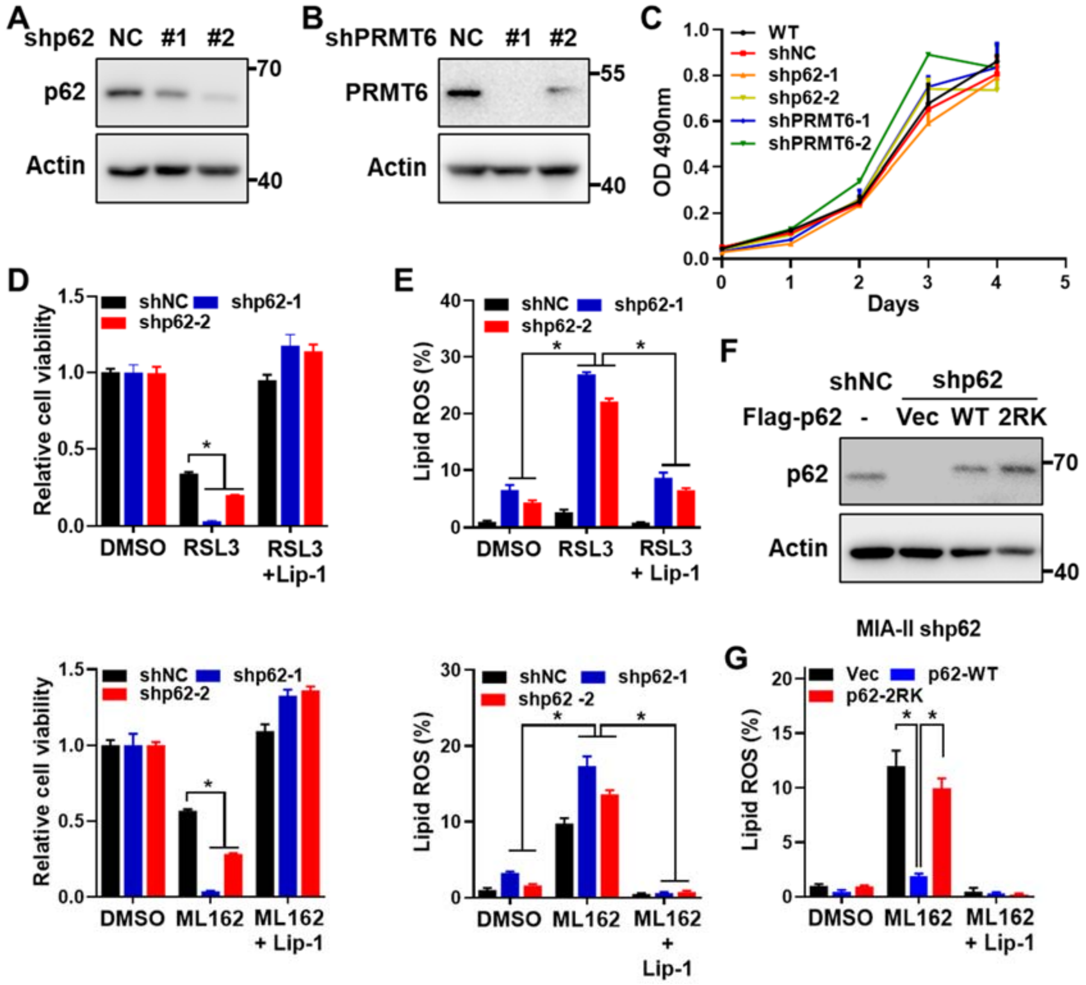

Figure S12. Stably knockdown of p62 or PRMT6 sensitized ferroptosis *in vitro*

A-B. Knockdown of p62 (A) or PRMT6 (B) in MIAPACA-II stable knockdown cells (shp62 or shPRMT6) was validated by western blotting.

C. Cell viability of parental MIAPACA-II cells or stable knockdown cells (shp62

or shPRMT6) was determined by MTS assay.

D. After MIAPACA-II shNC or shp62 cells were treated with 2 μM RSL3 (upper) or 1 μM ML162 (down) for 48 h, together with or without 0.25 μM Lip-1, cell viability was measured by MTS assay.

E. After MIAPACA-II shNC or shp62 cells were treated with 2 μM RSL3 (upper) or 1 μM ML162 (down) for 48 h, together with or without Lip-1, the lipid ROS was detected with C11-BODIPY reagent.

F. Flag-p62-WT or 2RK were introduced into MIAPACA-II shp62 (MIA-shp62) cells, and the protein level of Flag-p62-WT or 2RK was determined by western blotting with anti-p62.

G. After Flag-p62-WT or 2RK introduced into MIA-shp62 cells, 1 μM ML162 was added for 48 h, together with or without Lip-1, and the lipid ROS was detected with C11-BODIPY reagent.

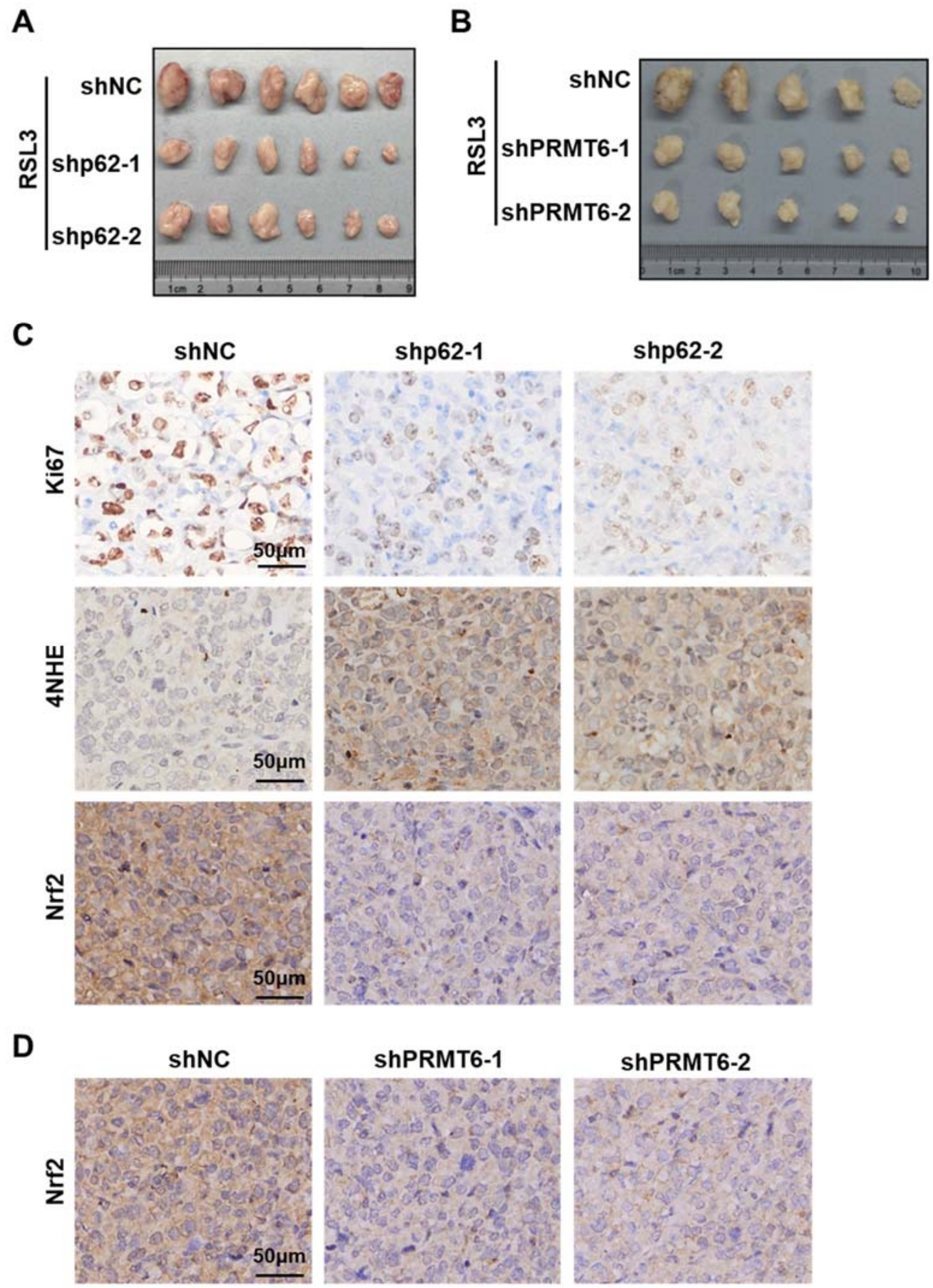

Figure S13. Inhibition of PRMT6-mediated p62 ADMA sensitized Ferroptosis in pancreatic cancer *in vivo*

A-B. The represented tumor pictures from MIA-shp62 (A) or MIA-shPRMT6 (B) xenograft experiments were shown.

C. MIA-shNC or MIA-shp62 xenograft tumor sections were stained with Nrf2, Ki67 and 4HNE by Immunohistochemistry (IHC).

D. MIA-shNC or MIA-shPRMT6 xenograft tumor sections were stained with Nrf2, by IHC.

Supplemental Table

Primers	
**Name**	**Sequences**
**HMOX1-F**	5-TTCAAGCAGCTCTACCGCTC-3
**HMOX1-R**	5-TTCAAGCAGCTCTACCGCTC-3
**NQO1-F**	5-GCACTGATCGTACTGGCTCA-3
**NQO1-R**	5-CGCAGGGTCCTTCAGTTTAC-3
**GFP-p62-F**	5-ATACTCGAGTGGCGTCGCTCACCGT-3
**GFP-p62-R**	5-TAGGATCCTCACAACGGCGGGGGAT-3
**mCherry-PRMT6-F**	5-AGTCCGGACTCAGATCTCGAGTGTCGCAGCCCAAGAAAAGA-3
**mCherry-PRMT6-R**	5-TTATCTAGATCCGGTGGATCCTCAGTCCTCCATGGCAAAGT-3
**p62-R183K-F**	5-TCACCTTCCGGAGCCATTTGCTGTGCGAGAAGCCC-3
**p62-R183K-R**	5-GGGCTTCTCGCACAGCAAATGGCTCCGGAAGGTGA-3
**p62-R217K-F**	5-GCCGTGGGGCCAGGTTTGGCCTCCCCTGCAC-3
**p62-R217K-R**	5-GTGCAGGGGAGGCCAAACCTGGCCCCACGGC-3
**p62-R183F-F**	5-TCACCTTCCGGAGCCAGGTGCTGTGCGAGAAGCCC-3
**p62-R183F-R**	5-GGGCTTCTCGCACAGCCCATGGCTCCGGAAGGTGA-3
**p62-R217F-F**	5-GCCGTGGGGCCAGGGGTGGCCTCCCCTGCAC-3
**p62-R217F-R**	5-GTGCAGGGGAGGCCCCACCTGGCCCCACGGC-3
**PRMT6mt-F**	5-CGAGGCAAGACGAAACTGGCCGTGGGCGCGGG-3
**PRMT6mt-R**	5-CCCGCGCCCACGGCCAGTTTCGTCTTGCCTCG-3
**p62-shRNA resis-F**	5-GTCCACTTCTTTTGAAGATAGGTGTGTCCAATCATCATCTCCT CCTGAACAGTTATC-3
**p62-shRNA resis-R**	5-GATAACTGTTCAGGAGGAGATGATGATTGGACACACCTATC TTCAAAAGAAGTGGAC-3
**SiRNAs/shRNAs**	
**Name**	**Sequences**
**sip62-1**	GUGACGAGGAAUUGACAAUTT
	AUUGUCAAUUCCUCGUCACTT
**sip62-2**	GGAGUCGGAUAACUGUUCATT
	UGAACAGUUAUCCGACUCCTT
**siPRMT6-1**	CCAGGUGAAGCAGCACUAUTT
	AUAGUGCUGCUUCACCUGGTT
**siPRMT6-2**	GAGUGCUACUCGGACGUUUTT
	AAACGUCCGAGUAGCACUCTT
**siNrf2-1**	GCAGUUCAAUGAAGCUCAACUTT
	AGUUGAGCUUCAUUGAACUGCTT
**siNrf2-2**	GCAUUGGAGUGUCAGUAUGTT

	CAUACUGACACUCCAAUGCTT
**shp62-1**	5-CCTCTGGGCATTGAAGTTGAT-3
**shp62-2**	5-ACTGGACCCATCTGTCTTCAA-3
**shPRMT6-1**	5-CACGGACGTTTCAGGAGAGAT-3
**shPRMT6-2**	5-GCGAACCAAGTGGCTGAAGGA-3
